# Difference in Efficacy and Safety of Anti-CD19 Chimeric Antigen Receptor T-Cell Therapy Containing 4-1BB and CD28 Co-Stimulatory Domains for B-Cell Acute Lymphoblastic Leukemia

**DOI:** 10.3390/cancers15102767

**Published:** 2023-05-15

**Authors:** Lijuan Wu, Junchao Chen, Ruifen Cai, Xinrui Wang, Yixiao Liu, Qingshan Zheng, Lujin Li

**Affiliations:** Center for Drug Clinical Research, Shanghai University of Traditional Chinese Medicine, No.1200 Cailun Road, Shanghai 201203, China; leahww@163.com (L.W.); jchen@shutcm.edu.cn (J.C.); 15768966773@163.com (R.C.); r884153@163.com (X.W.); liuyixiao516@163.com (Y.L.)

**Keywords:** co-stimulatory domain, anti-CD19 CAR T, anti-CD19 chimeric antigen receptor, B-cell acute lymphoblastic leukemia, model-based meta-analysis

## Abstract

**Simple Summary:**

To date, quantitative and comprehensive information summarizing the differences in the efficacy and safety of anti-CD19 chimeric antigen receptor (CAR) T-cells with CD28 co-stimulatory domains or 4-1BB co-stimulatory domains for the treatment of B-cell acute lymphoblastic leukemia (B-ALL) is lacking. Our study utilized a model-based meta-analysis (MBMA) to accurately measure the differences between the anti-CD19 CAR T-cell therapies for B-ALL with CD28 and 4-1BB co-stimulatory domains. We discovered that the median overall survival (OS) and progression-free survival (PFS) for the 4-1BB co-stimulatory domain were longer than those for anti-CD19 CAR T-cell therapy with a CD28 co-stimulatory domain. Additionally, anti-CD19 CAR T-cells with the CD28 co-stimulatory domain showed a higher incidence of neurotoxic adverse effects. This study provides quantitative information that can be used to compare the different co-stimulatory domains of anti-CD19 CAR T-cells for treating B-ALL.

**Abstract:**

This study quantified the differences in the efficacy and safety of different stimulation domains of anti-CD19 chimeric antigen receptor (CAR) T therapy for B-cell acute lymphoblastic leukemia (B-ALL). Clinical trials related to anti-CD19 CAR T-cell therapy for B-ALL were searched in public databases from database inception to 13 November 2021. The differences in overall survival (OS) and progression-free survival (PFS) of B-ALL patients treated with anti-CAR T-cell therapy containing 4-1BB and CD28 co-stimulatory domains were compared by establishing a parametric survival function. The overall remission rate (ORR), the proportion of people with minimal residual disease (MRD)-negative complete remission (CR), the incidence of cytokine release syndrome (CRS), and the neurotoxicity across different co-stimulatory domains was assessed using a random-effects model. The correlation between the ORR, MRD-negative CR, PFS, and OS was tested. The results showed that the median OS of anti-CAR T-cell treatment containing 4-1BB and CD28 co-stimulatory domains was 15.0 months (95% CI: 11.0–20.0) and 8.5 months (95% CI: 5.0–14.0), and the median PFS was 7.0 months (95% CI: 4.0–11.5) and 3.0 months (95% CI: 1.5–7.0), respectively. Anti-CD19 CAR T-cells in the 4-1BB co-stimulatory domain showed superior benefits in patients who achieved ORR. The incidence of neurotoxicity was significantly higher in the CD28 co-stimulatory domain of anti-CD19 CAR T-cells than in the 4-1BB co-stimulatory domain. In addition, the ORR and MRD-negative CR were strongly correlated with OS and PFS, and PFS and OS were strongly correlated. The 4-1BB co-stimulatory domain suggested a better benefit–risk ratio than the CD28 co-stimulatory domain in B-ALL.

## 1. Introduction

B-cell acute lymphoblastic leukemia (B-ALL) is a type of hematopoietic malignancy that originates from the abnormal proliferation and aggregation of lymphocytes in the bone marrow and/or in vitro [1]. It inhibits normal hematopoiesis, and is characterized by a rapid onset, high malignancy, and rapid metastasis [2]. If left untreated, B-ALL can become life threatening. Although most adult patients with B-ALL can go into remission with upfront chemotherapy, the relapse rate is as high as 50% [3,4]. For refractory and relapsed B-ALL, allogeneic hematopoietic stem-cell transplantation (allo-HSCT) is the ultimate treatment after remission, but the 5-year survival rate for this treatment is only 10% [5], and 32–50% of patients relapse [6]. Thus, there is still a huge unmet need for the clinical treatment of this disease.

In recent years, anti-CD19 chimeric antigen receptor (CAR) T-cell therapies have been widely used for hematologic tumors, among which anti-CD19 CAR T-cell therapies can achieve complete remission (CR) rates of 70–90% for B-ALL [7,8,9]. As various generations of CAR T-cells continue to be developed, their safety and efficacy have significantly improved. Presently, four generations of CAR T-cells are widely utilized in clinical practice [10]. First-generation CAR T-cells solely consist of an antigen-recognition monoclonal antibody and a signaling domain. Second- and third-generation CAR T-cells incorporate one or multiple co-stimulatory regions to enhance therapeutic efficacy and reduce adverse effects. The newest fourth-generation CAR T-cells employ transcription factors or other regulatory elements to augment their potency, representing a significant advance in the field. The US Food and Drug Administration (FDA) approval for CAR T-cell therapy is limited to second-generation CAR T-cell products. The CAR structure includes an antigen-specific single-chain variable fragment (scFv) outside the T-cell, hinge region, transmembrane region, CD3ζ T-cell activation region, and co-stimulatory region inside the T-cell, with the co-stimulatory region mainly derived from CD28 and 4-1BB [11].

The activation of natural T-cells involves two signals. The first signal is regulated by the CD3ζ T-cell activation region [12], which recognizes and binds to the major histocompatibility complex (MHC) on antigen-presenting cells (APCs) [13]. The second signal is controlled by the co-stimulatory region. The interaction between these receptors triggers the recruitment of signaling proteins and the induction of downstream signaling pathways within T-cells, leading to the activation of kinases [10,11]. As a result, the activated T-cells proliferate and differentiate into various types of effector T-cells and release cytokines to stimulate and regulate the immune response.

Previous meta-analyses have shown that different anti-CD19 CAR T-cell co-stimulatory structures do not differ significantly in terms of complete remission rate, 1-year progression-free survival (PFS), and 1-year overall survival (OS) [14]. A study showed that anti-CD19 CAR T-cells expressing different co-stimulatory structural domains had similar antitumor activity; however, CD28-co-stimulated CAR T-cells were more likely to cause neurotoxicity, but these findings were not robust and may have been confounded by multiple factors [13,15]. Notably, no large-scale clinical trials have compared the efficacy and safety of different anti-CD19 CAR T-cell co-stimulatory domains in the treatment of B-ALL.

Model-based meta-analysis (MBMA) is a comprehensive quantitative analysis of drug efficacy or safety by quantitative pharmacological modeling methods based on extensive literature data and is one of the important methods for strategic model-informed drug development [16,17]. Compared with traditional meta-analyses, MBMA can establish time effect models and covariate models to correct for inter-trial heterogeneity and make study conclusions more reliable. In this study, MBMA was used to establish a survival model of anti-CD19 CAR T-cell therapy for B-ALL and quantitatively analyze the distribution characteristics of different co-stimulatory structural domains on survival data and their influencing factors, with the aim of providing reliable quantitative information for anti-CD19 CAR T-cell therapy for B-ALL.

## 2. Methods

### 2.1. Search Strategy and Selection Criteria

A literature search was conducted in three public databases, namely PubMed, Embase, and Cochrane Library, to collect data and organize the literature according to the PRISMA reporting guidelines. The search deadline was 13 November 2021, and the search terms were “CAR T Cell”, “anti-CD19” and “B cell acute lymphoblastic leukemia” with entries in the same category connected by the logical operator “OR” and entries in different categories connected by the logical operator “AND”. The detailed search strategies are provided in Supplementary Materials Method S1. Additionally, a manual search was performed based on the references of relevant meta-analyses and reviews to add the missing literature.

The detailed method for the inclusion and exclusion criteria is shown in Supplementary Materials Method S2.

### 2.2. Data Extraction

The following data were extracted using Microsoft Excel (version 2016): (1) literature characteristics (literature DOI, authors, year of publication, clinical trial registration number, etc.), (2) trial design (sample size, dosing regimen, dose administered, co-stimulatory domains of CD19 CAR T-cells, etc.), (3) subject characteristics (median age, sex ratio, proportion of patients with primary refractory disease, burden of disease [median bone marrow blast percentage, percentage of patients with morphological relapse] etc.), and (4) outcome indicators (overall remission rate [ORR], overall survival, progression-free survival, incidence of adverse reactions, and incidence of grade 3/4 adverse reactions). If the outcome indicators were presented graphically in the literature, the Get Data Graph Digitizer (2.22) was used for data reading. The data extraction was performed by two investigators, and if the reading error was greater than 2%, the data were re-extracted and averaged for the final analysis.

### 2.3. Risk of Bias Assessment

Since all of the studies included were non-randomized observational studies, we assessed the risk of bias using the Risk of Bias in Non-randomized Studies of Interventions (ROBINS-I) tool [18]. This tool evaluated the certainty of evidence from seven domains, namely confounding, participant selection, intervention classification, deviations from intended interventions, missing data, outcome measurement, and reported result selection. Each study was evaluated independently by two researchers, and any discrepancies were resolved through discussions involving a third researcher.

### 2.4. Modeling Analysis of Overall and Progression-Free Survival

This study uses a parametric survival model to describe the OS and PFS data. Compared to semi-parametric survival models (such as the Cox proportional hazards regression model), parametric survival models can predict the entire process of changes in the OS and PFS over time. The process of building a parametric survival model involved creating structural effects, random effects, and covariate models. Detailed descriptions are shown in Supplementary Materials Method S3.

The model’s accuracy, predictability, sensitivity, and robustness were evaluated using Goodness of fit (GOF) plots, visual predictive check (VPC), leave-one-out cross-validation, and bootstrap methods. Additional information can be found in Supplementary Materials Method S4.

Using the final model, Monte Carlo simulation was performed 1000 times to calculate the typical value and 95% confidence interval (CI) of the OS and PFS for patients with B-ALL who were treated with anti-CD19 CAR T-cell therapy with different co-stimulatory domains. Additionally, we performed a subgroup analysis to investigate the potential factors that could influence the OS and PFS, regardless of whether they were considered in the covariate model. These factors included the age group (pediatric vs. adult), the proportion of patients with primary refractory B-ALL, bridging allo-HSCT after CAR T-cell therapy (percentage of treated population share), CD19 single-chain variable fragment clone (FMC63 vs. others), T-cell origin (autologous vs. allogeneic), and the sample size of trials. The subgroup analysis had two steps. In the first step, the estimated values of the model parameters and their standard errors were obtained for each study. Then, the original individual estimates of the model parameters were adjusted by the inverse calculation of the covariate functions to determine the varying degrees of covariate impact across different studies. In the second step, the estimated model parameters were pooled under these predefined subgroups using a random effects meta-analysis of a single mean to obtain the overall mean and 95% CI. Based on the distribution of parameters in each subgroup, 1000 Monte Carlo simulations were conducted to estimate the distribution of the OS and PFS in each subgroup.

### 2.5. Meta-Analysis for the Secondary and Safety Outcomes

The random effects model in the single-arm meta-analysis was used to analyze the ORR and the proportion of patients achieving minimal residual disease (MRD)-negative CR, the incidence of cytokine release syndrome (CRS), and neurotoxicity in patients with B-ALL treated with anti-CD19 CAR T-cell therapy with different co-stimulatory domains.

In addition, the subgroup analysis was used to calculate the ORR, incidence of CRS, and neurotoxicity under different factors, including a CD19 single-chain variable fragment clone (FMC63 vs. others), T-cell origin (autologous vs. allogeneic), age group (pediatric vs. adult), percentage of patients with primary refractory B-ALL, disease burden (percentage of morphologic relapse in patient population), and subsequent allo-HSCT bridging allo-HSCT after CAR T-cell therapy (the percentage of treated population share).

### 2.6. Correlation between ORR, MRD-Negative CR, PFS, and OS

Investigating the correlation between the clinical endpoint OS and potential short-term outcome of interest, such as ORR, MRD-negative CR, PFS, or EFS [19,20], can accelerate the development of new drugs. This approach has been identified in several malignant hematological tumors [21], we similarly assessed the correlation in the included data.

Weighted linear regression analysis was used to assess the correlation between ORR, MRD-negative CR, PFS, and OS: (1) 0.5- and 1-year PFS, (2) 1- and 2-year OS, (3) ORR, and (4) MRD-negative CR. The Pearson correlation coefficient (R^2^) was used to measure the strength of the correlation between the efficacy indicators. We considered a prespecified R^2^ value of ≥0.8 to indicate a high correlation, 0.6–0.8 to indicate a moderate correlation, and <0.6 to indicate a low correlation.

### 2.7. Software and Statistical Analysis

The modeling and simulation processes for this study were conducted using NONMEM 7.4 (ICON plc, Warrington, PA, USA). The first-order conditional estimation method (FOCE-I) was selected for estimating the model parameters. Meta-analysis and graphical visualization were performed using R 4.1.1. Several R packages, including meta, metafor, tidyverse, ggplot2, multiplot, and forestplot, were used for conducting the meta-analysis and generating the graphical visualizations.

## 3. Results

### 3.1. Characteristics of the Included Studies

A total of 336 studies were retrieved from the PubMed, EMBASE, and Cochrane Library databases, and 13 studies were supplemented from the references of relevant meta-analyses. The detailed search strategy is described in Supplementary Materials Table S1. According to the inclusion criteria, 31 publications with 928 subjects were finally included [7,8,9,22,23,24,25,26,27,28,29,30,31,32,33,34,35,36,37,38,39,40,41,42,43,44,45,46,47,48,49]. The literature screening process is shown in Supplementary Materials Figure S1. All included studies were second-generation CAR T-cell therapies, of which 26 of the 31 publications included anti-CD19 CAR T studies with the 4-1BB co-stimulatory domain (*N* = 700) and five studies with the CD28 co-stimulatory domain (*N* = 228). A list of the included studies and their information is shown in Supplementary Materials Tables S5–S7.

The average age of the participants was 6.5–53.6 years (median, 29.5 years), 37.5–92.9% (median, 57.3%) were male, and the mean number of treatments was 1–9 times (median, 3). The proportion of patients with primary refractory B-ALL was 0–55.0% (median, 10.6%). The median bone marrow blasts, proportion of morphologic relapses, and proportion of MRD-positive CR ranged from 4.0% to 74.0% (median, 49.0%), 28.6% to 100.0% (median, 82.5%), and 0.0% to 62.5% (median, 10.0%), respectively. The proportions of allo-HSCT before and after treatment with anti-CD19 CAR T-cells were 0.0–100.0% (median, 46.0%) and 0.0–82.6% (median, 21.7%), respectively. The baseline patient characteristics are shown in Supplementary Materials Table S4.

According to the ROBINS-I tool, the risk of bias was rated as low in eight studies (25.8%), moderate in 19 (61.3%), and serious in five (12.9%; Supplementary Materials Table S8, Figure S2).

### 3.2. Model Establishment and Evaluation

Based on an objective function value (OFV) minimization and the precision of the model parameter estimation, the log-normal hazard function was selected as the basic hazard function model to describe the OS and PFS data. The detailed OS and PFS model exploration processes are described in Supplementary Materials Tables S2 and S3. Covariate analysis showed that the proportion of morphological relapse had a significant impact on both the OS and PFS models. The covariate model can be expressed as follows:

(1) $$\mathrm{OS}\;\mathrm{model}:\;h{\left(t\right)}_{final}=h{\left(t\right)}_{base}\times e^{\left(COV-0.85\right)\times 1.22}$$

(2) $$\mathrm{PFS}\;\mathrm{model}:\;h{\left(t\right)}_{final}=h{\left(t\right)}_{base}\times e^{\left(COV-0.85\right)\times 1.78}\;$$

In Equations (1) and (2), *COV* represents the proportion of morphological relapse, and 0.85 is the median proportion of morphological relapse.

The final parameters of the model are presented in Table 1. The results show that the structural parameters σ and μ in the final models for OS and PFS can be classified into two categories based on the co-stimulatory domain, with differences observed in the same parameter among co-stimulatory domains (μ: 2.83 vs. 2.23 [OS] and 2.13 vs. 1.56 [PFS]). The included covariate model parameter is θ_Morphological relapse_, estimated to be 1.22 and 1.78 in the OS and PFS models, respectively. This suggests that for every 10% increase in the proportion of morphological recurrences, the risk of death and disease progression will increase by 13% (exp [1.22–0.1]–1) and 19.5% (exp [1.78–0.1]–1), respectively. The median of the parameters of the Bootstrap process is similar to the estimated value of the parameters in the original dataset, indicating that the estimated value of the model is robust. Moreover, the shrinkage values of the variability parameters are all less than 20%, indicating that the modeling data are sufficiently rich for the parameter estimation. Furthermore, Table 1 shows that 95.9% and 91.9% of the bootstrap runs were successful for the OS and PFS models, respectively, indicating that the models were stable. Supplementary Materials Figures S3–S6 display the GOF and Individual fit plots of the final model for the OS and PFS, indicating that the model fits the observations well without significant bias. The sensitivity analysis results of the parameters reveal that the estimation results of the model parameters are robust and are not affected by any study (Supplementary Materials Figure S7). The VPC plot indicates that the model has a good predictive power (Supplementary Materials Figure S8).

### 3.3. Typical Overall and Progression-Free Survival Simulation

**Figure 1:**
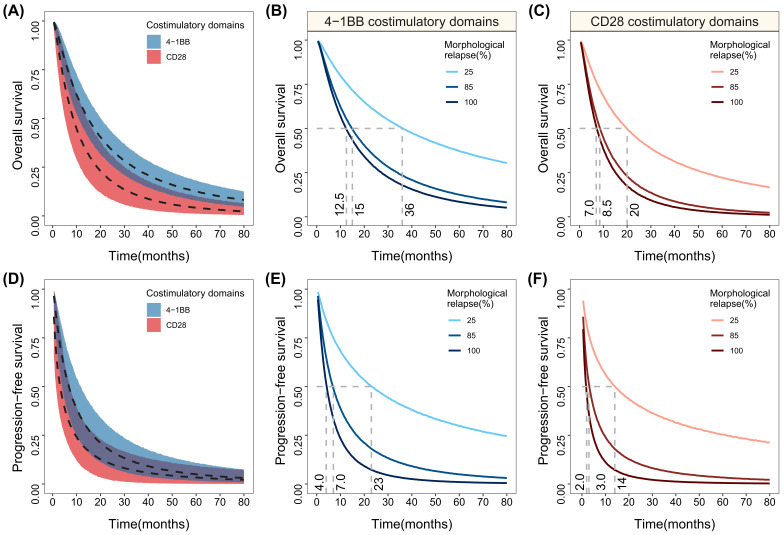
The simulation of the typical response distributions of OS and PFS in different co-stimulatory domains and their distribution at different levels of impact covariates. (**A**,**D**) The typical response distributions of different co-stimulatory domains OS and PFS. (**B**,**E**) The simulated distribution of 4-1BB co-stimulatory domain anti-CD19 CAR T at different levels of morphological relapse proportions. (**C**,**F**) The simulated distribution of CD28 co-stimulatory domain anti-CD19 CAR T at different levels of morphological relapse. The ribbon area is the 95% percentile line of the model simulation, and the solid and dashed curves in the figure are the median value lines.

Based on the final model, the typical OS and PFS and the 95% CIs of anti-CD19 CAR T-cell therapy with 4-1BB and CD28 co-stimulatory signals were simulated. The results (Table 2, Figure 1A) show that when the morphologic relapse rate is adjusted to 85% (median), the median OS of 4-1BB and CD28 co-stimulatory domain was 15.0 months (95% CI: 11.0–20.0) and 8.5 months (95% CI: 5.0–14.0), respectively, and the former was 6.5 months longer than the latter, with a partial overlap of the confidence intervals. The 1-year overall survival rates of the 4-1BB and CD28 co-stimulatory domains were 56.6% (95% CI: 47.1–67.6%) and 38.6% (95% CI: 24.0–56.6%), respectively, and the former was 18.0% higher than the latter. This study found that the morphologic relapse rate significantly affected the OS. When the morphologic relapse rate was 25% and 100%, the median OS of the 4-1BB-co-stimulated CAR T-cells was 36 and 12.5 months respectively, and the median OS of the CD28-co-stimulated CAR T-cells was 20 and 7 months, respectively (Figure 1B,C).

The median PFS for the 4-1BB and CD28 co-stimulatory domains (Table 2, Figure 1D) was 7.0 months (95% CI: 4.0–11.5) and 3.0 months (95% CI: 1.5–7.0), respectively; the former was 4 months longer than the latter, with a partial overlap of the confidence intervals. The 1-year PFS rates of the 4-1BB and CD28 co-stimulatory domains were 33.8% (95% CI: 20.7–49.6%) and 20.8% (95% CI: 9.0–34.8%), respectively; the former was 13.0% higher than the latter. When the morphologic relapse rate was 25% and 100%, the median PFS of the 4-1BB-co-stimulated CAR T-cells was 23 and 4 months, respectively, and the median PFS of the CD28-co-stimulated CAR T-cells was 14 and 2 months, respectively (Figure 1E,F).

**Figure 2:**
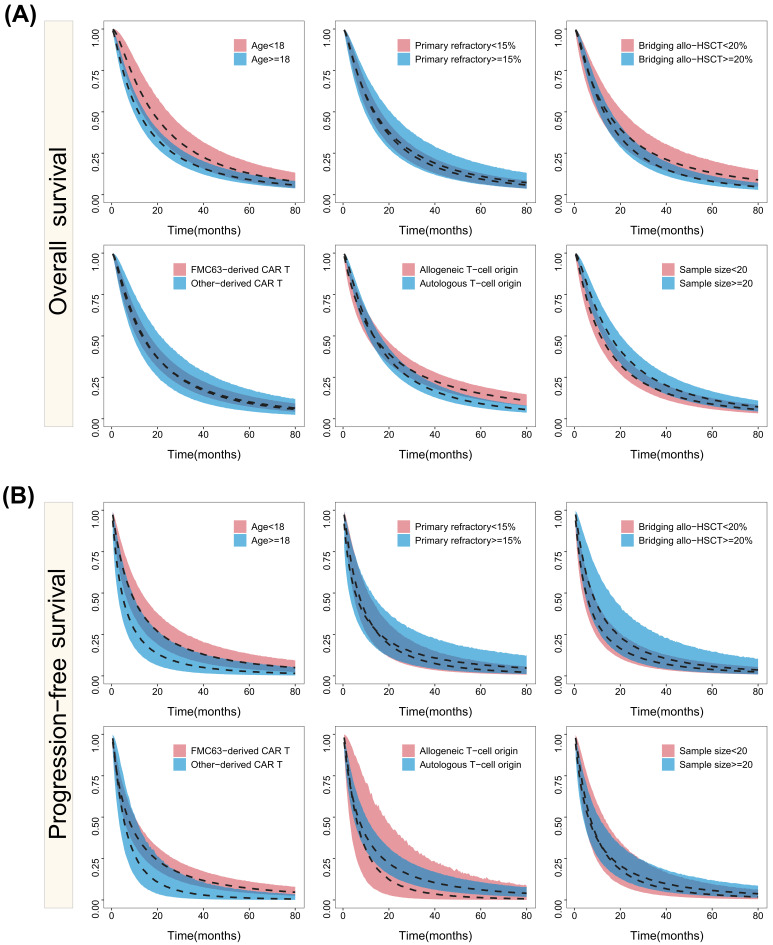
The simulated survival results and their 95% CIs of the OS (**A**) and PFS (**B**) of the subgroup analyses. The ribbon area is the 95% percentile line of the model simulation and the dashed line is the median value line and different colors represent different subscale levels.

Figure 2 depicts the results of the subgroup analysis, suggesting a potential correlation between the age and OS and PFS. Although the 95% CIs for the OS and PFS partially overlap for both age groups above and below 18 years old, the observed trend indicates a slower decrease in the OS and PFS among patients under 18 years old. However, no trends were found in other subgroups, including the proportion of patients with primary refractory B-ALL (<15% vs. ≥15%), the proportion of bridging allo-HSCT after CAR T-cell therapy (<20% vs. ≥20%), CD19 single-chain variable fragment clone (FMC63 vs. others), T-cell origin (autologous vs. allogeneic), and the sample size of trials (<20 vs. ≥20).

### 3.4. Meta-Analysis for Secondary and Safety Outcomes

**Figure 3:**
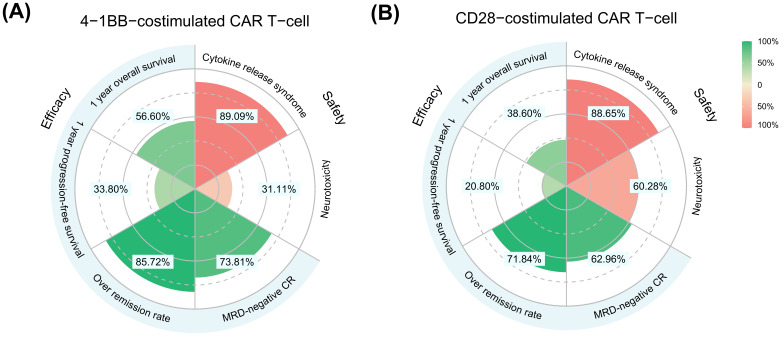
4-1BB-co-stimulated (**A**) and CD28-co-stimulated (**B**) CAR-T-cell efficacy and safety summary. Efficacy (**left**) reports 1-year overall survival, 1-year progression-free survival, overall remission rate, and minimal residual negative disease complete remission rate. Safety (**right**) reports the rates of cytokine release syndrome and neurotoxicity. The colors indicate percentages, from green (efficacy), to red (safety). The estimated event rates are indicated by text within light blue rectangles. MRD, minimal residual disease; CR, complete remission.

Based on the analysis of different co-stimulatory domains, the results (as shown in Figure 3 and Supplementary Materials Figure S9) indicated that the ORR of the 4-1BB co-stimulatory domain and CD28 co-stimulatory domain was 85.72% and 71.84%, respectively, and the proportion of MRD-negative CR was 73.81% and 62.96%, respectively. In terms of safety, the incidences of CRS in the 4-1BB and CD28 co-stimulatory domains were 89.09% and 88.65%, respectively. The incidence of grade 3 and 4 CRS was 29.54% and 23.45%, respectively. There were no significant differences between the above outcomes, except for the ORR, and the percentage of patients achieving ORR with anti-CAR T-cell therapy in the 4-1BB co-stimulatory domain was superior to that in the CD28 co-stimulatory domain. In addition, the incidence of neurotoxicity and grade 3–4 neurotoxicity was significantly higher in the CD28-co-stimulated CAR T-cells than in the 4-1BB-co-stimulated CAR T-cells (60.28% vs. 31.11% and 28.31% vs. 7.78%, respectively).

In addition to the co-stimulatory domain, the subgroup analyses were performed on the effects of scFv type, T-cell origin, age, the proportion of the primary refractory population, the proportion of the morphologically relapsed population, and the proportion of the population receiving allo-HSCT after CAR T-cell therapy on ORR and adverse effects (Supplementary Materials Figures S10–S12). The results showed a trend towards a higher ORR in children than in adults, with 86.59% and 76.32%, respectively, a trend towards a higher ORR in the low morphologic relapse population than in the high morphologic recurrence relapse population, with 85.54% and 74.08%, respectively, and a trend towards a lower incidence of CRS and neurotoxicity in allogeneic T-cells than in autologous T-cells, with 72.00% vs. 88.66% and 41.4% vs. 21.47%, respectively; however, none of these results showed a significant difference because their 95% CIs mostly overlapped.

### 3.5. Correlation between ORR, MRD-Negative CR, PFS, and OS

**Figure 4:**
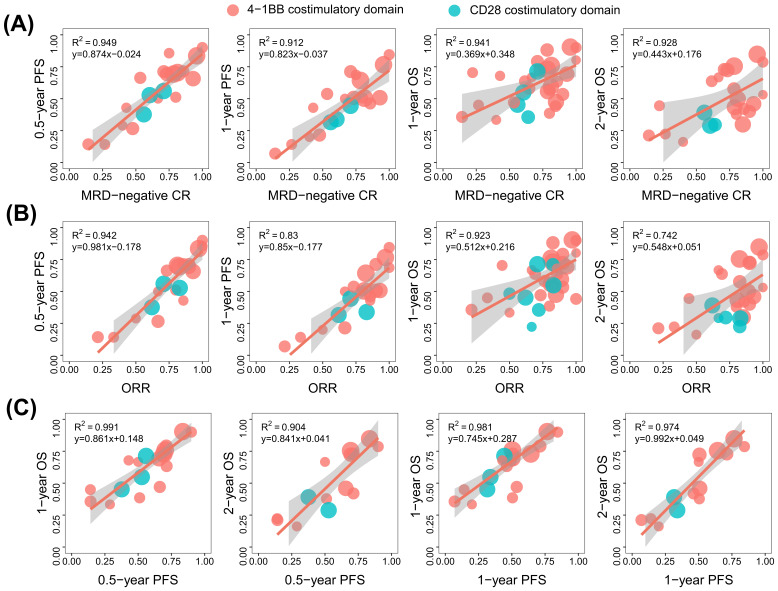
Correlation between ORR, MRD-negative CR, PFS, and OS (**A**–**C**).

Figure 4 shows the correlations between the ORR, MRD-negative CR, PFS, and OS. The results (Figure 4A) indicate that MRD-negative CR showed a high correlation with 0.5- and 1-year PFS and 1- and 2-year OS with a Pearson coefficient R^2^ of 0.949, 0.912, 0.941, and 0.928, respectively. In addition, the ORR showed a high correlation with 0.5- and 1-year PFS and 1-year OS (Figure 4B) with a Pearson coefficient R^2^ of 0.942, 0.83, and 0.923, respectively, but the ORR showed a moderate correlation with 2-year OS with a coefficient R^2^ of 0.742. Additionally, the 0.5- and 1-year PFSs were highly correlated with 1- and 2-year OS, with a Pearson coefficient R^2^ of 0.991, 0.904, 0.981, and 0.974, respectively (Figure 4C).

The red points are the 4-1BB co-stimulated anti-CD19 CAR T group, the green points are the CD28 co-stimulated domain group, the point size is proportional to the number of patients in each trial, the red solid line is the weighted regression line, and the shaded area represents its 95% CI.

## 4. Discussion

At present, there are no clinical trials with a large sample size to compare the efficacy and safety of 4-1BB and CD28 co-stimulatory domains in second-generation CAR T-cell therapy. The conclusions reported in previous studies are inconsistent and contradictory. For example, a clinical trial [15] reported that 10 patients received anti-CD19 CAR T-cells with CD28 and 4-1BB co-stimulatory domains. Although the two types of CAR T-cells were effective, they showed different reaction patterns and adverse effects. A meta-analysis^14^ showed that although there was no difference in the ORR, 1-year OS, and 1-year PFS among different co-stimulatory domains of anti-CD19 CAR T-cell therapy, patients who received the 4-1BB co-stimulatory domain had a higher proportion of MRD-negative CR than those who received the CD28 co-stimulatory domain. A retrospective study [50] combining several clinical trials found that the factors associated with EFS at *p* < 0.1 were CAR construct type, and 4-1BB co-stimulatory domains were superior to CD28 co-stimulatory domains. However, a preclinical study [51] showed that CAR T-cells with a CD28 co-stimulatory domain have a stronger anti-tumor response, and that CAR T-cells with a 4-1BB co-stimulatory domain take longer to achieve similar effects.

Based on extensive literature data, this study quantitatively compared the efficacy and safety of anti-CD19 CAR T-cell therapy including the 4-1BB and CD28 co-stimulatory domains in the treatment of B-ALL for the first time. The results showed while there was some overlap in the 95% CIs of the OS and PFS, the overall trend indicated that the effect of anti-CD19 CAR T-cell therapy with the 4-1BB co-stimulatory domain was better than that with the CD28 co-stimulatory domain. Similarly, this study found that the ORR and proportion of MRD-negative CR in 4-1BB-co-stimulated CAR T-cells were better than those in CD28-co-stimulated CAR T-cells. In terms of safety, the incidence of CRS in different co-stimulatory domains was similar, but the incidence of neurotoxicity in the CD28 co-stimulatory domain was significantly higher than that in the 4-1BB co-stimulatory domain. These results suggest that the 4-1BB co-stimulatory domain has a higher benefit–risk ratio than the CD28 co-stimulatory domain.

This study found that the proportion of morphological relapse had a significant effect on OS and PFS. For every 10% increase in the proportion of morphological relapses, the risk functions in the OS and PFS models increased by 13% and 19%, respectively. In B-ALL, a morphologic relapse was defined as ≥5% bone marrow blasts, the presence of circulating leukemia, or disease progression. Bone marrow blasts correlate with the disease burden of B-ALL, with higher bone marrow blast values associated with a higher disease burden [50]. This study showed that the lower the percentage of morphologic relapse in the population, the longer the median OS and PFS; that is, the lower the disease burden and the better the outcome. This study confirms previous data [9,24,37] demonstrating that patients with a low burden experience superior long-term outcomes following anti-CD19 CAR T-cell therapy compared to heavily pretreated patients with high-burden disease. The results mentioned above indicate that reducing the burden of tumor disease is essential for treating B-ALL. Therefore, selecting the appropriate chemotherapeutic agents and determining the extent of lymphatic depletion before administering anti-CD19 CAR T-cell therapy should be carried out carefully. The current study’s findings offer further proof that minimizing the disease burden before initiating CAR T-cell treatment can have a positive impact on the response to therapy.

We have not yet included some factors as covariates in the model because the reduction in the OFV has not reached the threshold. However, these factors are believed to potentially impact the OS, PFS, and ORR. Therefore, we conducted a subgroup analysis to explore the trend of their impact on the OS, PFS, and ORR. It should be noted that due to the lack of strict randomization, there may be confounding factors, so the results of the subgroup analysis need to be interpreted with caution. When exploring the potential impact factors through subgroup analyses of the OS and PFS, this study discovered a possible association between age and both outcomes. However, the 95% CIs for age showed a partial overlap between the subgroups. The study suggested a trend indicating that patients aged <18 years had a better median survival for both the OS and PFS compared to those aged ≥18 years, by 6 and 4 months, respectively. These findings are consistent with previous studies that suggest children have a higher response to treatment compared to adult patients [8,9]. Regarding the prognostic factors in acute lymphoblastic leukemia, an age between 1 and 10 years was considered a favorable prognostic factor [52]. On the other hand, adolescents and adults had a higher prevalence of high-risk molecular abnormalities in acute lymphoblastic leukemia and fewer low-risk biological subtypes, and were less tolerant to chemotherapy treatment than children [53].

When conducting subgroup analyses to investigate the influencing factors of ORR and adverse events, a trend was observed, suggesting that age and the proportion of morphological recurrences may impact ORR, which is consistent with the aforementioned results. Furthermore, the source of T-cells may have influenced the incidence of adverse events, with allogeneic sources having lower rates of CRS and neurotoxicity than autologous sources. However, the small sample sizes in the CRS incidence subgroup analysis (842 and 86 for autologous and allogeneic T-cell sources, respectively) and in the neurotoxicity incidence subgroup analysis (737 and 56 for autologous and allogeneic T-cell sources, respectively) may have contributed to the bias in the incidence of adverse events.

Overall survival is the most dependable clinical endpoint used to evaluate antineoplastic agents. However, since it requires the long-term follow-up of large populations and can be influenced by subsequent drugs, researchers seek valid or potential clinical endpoints to expedite the development of new clinical agents. In an analysis of long-term survival over 4 years [54], patients without EFS events at 12 and 24 months were found to have a longer OS than those with events at these time points. This study found a strong correlation between the MRD-negative CR and ORR, PFS, and OS, and a strong correlation between PFS and OS. These findings suggest that in the treatment of relapsed/refractory B-ALL, the treatment benefit of PFS can be translated into a benefit of OS. Short-term MRD-negative CR and ORR can predict PFS, and the 0.5- and 1-year PFS can be potential surrogate endpoints for 1- and 2-year OS. These endpoints may assist researchers in making prompt scientific decisions. Similar findings were reported in a meta-analysis [55], where MRD-negative CRs were associated with 10-year EFS (event-free survival). MRD-negative adults had a 10-year EFS of approximately 64%, compared to only 21% for MRD-positive adults, suggesting that the benefits for short-term endpoints such as MRD-negative contribute to long-term endpoint benefits.

Several studies have shown that after remission is achieved by CAR T-cell therapy, remission can be further maintained by HSCT, and this therapy is significantly associated with a low relapse rate [8,9]. In this study, the proportion of patients who received HSCT after anti-CD19 CAR T-cell therapy ranged from 0% to 82.6%. However, this study did not find that the proportion of HSCT treatments had a significant impact on the OS and PFS. This may be related to the fact that the design of the trial included in the study did not consider HSCT as the research factor.

It should be noted that there was heterogeneity in the criteria for assessing the CRS and neurotoxicity among different studies. There were at least two CRS assessment scales in the included studies (Supplementary Materials Table S7). A study [56] that assessed different criteria for the CRS found that only 25% of patients had the same CRS grade in each system for CRS, as different systems score symptoms differently, resulting in inconsistent final grades.

In addition to the aforementioned limitations, this study included a very small number of Ph+ B-ALL patients, which precluded further comparison for this subgroup. A study [50] investigating sequential CD19 targeting reported a lower rate of complete remission in responding patients who received anti-CD19 CAR T compared to non-responders treated with blinatumomab (64.5% vs. 93.5%). Unfortunately, our study did not analyze the impact of prior blinatumomab treatment on the efficacy of anti-CD19 CAR T-cell therapy due to the same sample size limitation. The data for this analysis were obtained from the literature, and some factors that may impact efficacy, such as race, prior receipt of other antibody treatments (e.g., Inotuzumab ozogamicin), extramedullary disease, and baseline CNS disease, were missing from over 30% of the studies and could not be tested as covariates. Additionally, this study did not consider dose heterogeneity in the treatment regimens due to data limitations. Finally, our study only included English-language publications, which may have introduced publication bias.

## 5. Conclusions

This study comprehensively compared the efficacy and safety of the 4-1BB and CD28 co-stimulatory domains of anti-CD19 CAR T-cells in the treatment of B-ALL. The results suggested that the OS and PFS of the 4-1BB co-stimulatory domain were longer than those of the CD28 co-stimulatory domain in CAR T-cell therapy, and this trend was also demonstrated in the ORR and MRD-negative CR. In terms of safety, the incidence of neurotoxicity was significantly higher in the CD28 co-stimulatory domain than in the 4-1BB CAR T-cell co-stimulatory domain. This study provides quantitative information for the comparison of different co-stimulatory domains of anti-CD19 CAR T-cells for the treatment of B-ALL.

## Figures and Tables

**Table 1:** Parameter estimates of the final model for the OS and PFS.

Parameter (Co-Stimulatory Domain)	OS Model				PFS Model			
	Final Model		Bootstrap (959/1000 *)		Final Model		Bootstrap (919/1000 *)	
	Value (RSE%)	Shrinkage (%)	Median	95% CI	Value (RSE%)	Shrinkage (%)	Median	95% CI
Survival parameters								
σ (4-1BB)	1.52 (7.8)		1.51	1.29–1.77	1.67 (8.9)		1.67	1.40–2.00
σ (CD28)	1.42 (10.5)		1.44	1.13–1.74	2.03 (24.4)		2.17	1.12–5.00
μ (4-1BB)	2.83 (5.1)		2.80	2.54–3.12	2.13 (12.5)		2.16	1.63–2.79
μ (CD28)	2.23 (11.1)		2.25	1.45–2.87	1.56 (20.9)		1.59	0.90–4.00
θ_Morphological relapse_	1.22 (43.4)		1.30	0.44–2.61	1.78 (21.6)		1.80	0.56–2.87
Variability parameters								
η (σ)	0.329 (13.6)	9.6	0.312	0.224–0.404	0.330 (14.4)	10.7	0.301	0.162–0.423
η (μ)	0.240 (13.7)	3.0	0.240	0.170–0.327	0.459 (15.0)	2.4	0.432	0.223–0.560
ε	0.623 (9.3)	6.7	0.616	0.501–0.725	0.727 (10.0)	6.9	0.729	0.574–0.858

η, inter-trial variability of model parameters; ε, residual error; CI, confidence interval; RSE, relative standard error. * The success rates of convergence for 1000 iterations of the Bootstrap method are 95.9% for the OS model and 91.9% for the PFS model.

**Table 2:** Predicted typical values and 95% CIs of OS and PFS in various co-stimulatory domains.

	4-1BB	CD28
Overall survival		
Median OS, month	15.0 (11.0–20.0)	8.5 (5.0–14.0)
1-year OS rate, %	56.6 (47.1–67.6)	38.6 (24.0–56.6)
2-year OS rate, %	34.8 (26.6–43.9)	18.3 (8.7–31.6)
5-year OS rate, %	12.5 (7.9–18.3)	4.2 (1.1–10.2)
Progression-free survival		
Median PFS, month	7.0 (4.0–11.5)	3.0 (1.5–7.0)
1-year PFS rate, %	33.8 (20.7–49.6)	20.8 (9.0–34.8)
2-year PFS rate, %	17.3 (8.8–29.0)	10.5 (2.1–19.8)
5-year PFS rate, %	5.0 (1.7–10.9)	3.4 (0.1–9.4)

## Data Availability

All studies included in this manuscript are accessible through Medline, Embase, and the Cochrane Library. Any data contained within this article are available upon request from the corresponding author.

## Supplementary Materials

The following supporting information can be downloaded at: https://www.mdpi.com/article/10.3390/cancers15102767/s1, Method S1: Full literature search strategy; Method S2: Inclusion/exclusion criteria; Method S3: Model building; Method S4: Model assessment; Table S1: Search strategy; Table S2: The process of exploring the structural model of OS; Table S3: The process of exploring the structural model of PFS; Table S4: Baseline characteristics of patients with B-cell acute lymphoblastic leukemia treated with anti-CD19 CAR T cells; Table S5: The list of included studies; Table S6: The details of the included studies; Table S7: Secondary and safety outcomes included in the studies; Table S8: Risk assessment for inclusion in the studies; Figure S1: Flow chart of study selection; Figure S2: The Summary risk of Literature assessment; Figure S3: The goodness-of-fit plots of OS model; Figure S4: The goodness-of-fit plots of PFS model; Figure S5: Individual trial fitting graph of OS model; Figure S6: Individual trial fitting graph of PFS model; Figure S7: Sensitivity analysis of model parameters by the leave-one-out cross validation; Figure S8: Visual prediction check of the final model of OS (A) and PFS (B); Figure S9: Comparison of different costimulatory domains in secondary and safety outcomes forest plot; Figure S10: Subgroup analysis of ORR outcome; Figure S11: Subgroup analysis of CRS outcome; Figure S12: Subgroup analysis of neurotoxicity outcome. References [7,8,9,22,23,24,25,26,27,28,29,30,31,32,33,34,35,36,37,38,39,40,41,42,43,44,45,46,47,48,49] are cited in the Supplementary Materials.

## Abbreviations

allo-HSCT: allogeneic hematopoietic stem-cell transplantation; APC: antigen-presenting cells; B-ALL: B-cell acute lymphoblastic leukemia; CAR: chimeric antigen receptor; CI: confidence in-terval; CR: complete remission; CRS: cytokine release syndrome; CWRES: conditional weighted residual errors; GOF: Goodness of fit; IL-2: interleukin-2; IPRED: individual prediction; MBMA: model-based meta-analysis; MRD: minimal residual disease; OBS: observation; OFV: objective function value; ORR: overall remission rate; OS: overall survival; PFS: progression-free survival; PRED: population prediction; ROBINS-I: Risk of Bias in Non-randomized Studies of Interventions; scFv: single-chain variable fragment; VPC: visual predictive check.
