# Supplementary material for: Difference in Efficacy and Safety of Anti-CD19 Chimeric Antigen Receptor T-Cell Therapy Containing 4-1BB and CD28 Co-Stimulatory Domains for B-Cell Acute Lymphoblastic Leukemia
Source: Cancers (Basel). 2023 May 15;15(10):2767. doi: 10.3390/cancers15102767 (PMC10216493; doi:10.3390/cancers15102767)
Supplement: Supplementary file 1 [file cancers-15-02767-s001.zip › cancers-2359407-supplementary.pdf]

Supplementary Materials

Method S1. Full literature search strategy.

Method S2. Inclusion/exclusion criteria.

Method S3. Model building.

Method S4. Model assessment.

Table S1. Search strategy.

Table S2. The process of exploring the structural model of OS.

Table S3. The process of exploring the structural model of PFS.

Table S4. Baseline characteristics of patients with B-cell acute lymphoblastic leukemia treated with anti-CD19 CAR T cells.

Table S5. The list of included studies.

Table S6. The details of the included studies.

Table S7. Secondary and safety outcomes included in the studies.

Table S8. Risk assessment for inclusion in the studies.

Figure S1. Flow chart of study selection.

Figure S2. The Summary risk of Literature assessment.

Figure S3. The goodness-of-fit plots of OS model.

Figure S4. The goodness-of-fit plots of PFS model.

Figure S5. Individual trial fitting graph of OS model.

Figure S6. Individual trial fitting graph of PFS model.

Figure S7. Sensitivity analysis of model parameters by the leave-one-out cross validation.

Figure S8. Visual prediction check of the final model of OS (A) and PFS (B).

Figure S9. Comparison of different costimulatory domains in secondary and safety outcomes forest plot.

Figure S10. Subgroup analysis of ORR outcome.

Figure S11. Subgroup analysis of CRS outcome.

Figure S12. Subgroup analysis of neurotoxicity outcome.

Method S1. Full literature search strategy

We conducted a comprehensive search of PubMed/MEDLINE, EMBASE, and Cochrane Library databases until November 13, 2021, using keywords such as "CAR T Cell", "anti-CD19", "B cell acute Lymphoblastic Leukemia", and similar search terms. The entries in the same category were connected with the logical word "OR", while the entries in different categories were linked with the logical word "AND". The complete search strategy is provided below. We downloaded the citations in Endnote X9, screened for duplicates, and manually excluded them using Endnote X9. Two researchers participated in the literature inclusion process, reading the full text of potential studies and creating tables for data extraction.

Table S1. Search Strategy.

No.	Query	Results
PubMed		
#1	(chimeric antigen receptor) OR (CAR T Cell) OR (CAR T-Cell) OR (CAR T) OR (Therapies, CAR T-Cell) OR (Therapy, CAR T-Cell) OR (T-Cell Therapy, CAR)	21918
#2	(anti-CD19) OR (anti CD19) OR (anti cd19) OR (CD19)	13398
#3	(B cell acute lymphocytic leukaemia) OR (B cell acute Lymphoblastic Leukemia) OR (B-ALL) OR (B cell Leukemia, Lymphoblastic, Acute) OR (Leukemia, B Cell)	46147
#4	#1 AND #2 AND #3	680
#5	#4 in Clinical Trial	70
Cochrane Library		
#1	(chimeric antigen receptor) OR (CAR T Cell) OR (CAR T-Cell) OR (CAR T) OR (Therapies, CAR T-Cell) OR (Therapy, CAR T-Cell) OR (T-Cell Therapy, CAR)	2937
#2	(anti-CD19) OR (anti CD19) OR (anti cd19) OR (CD19)	943
#3	(B cell acute lymphocytic leukaemia) OR (B cell acute Lymphoblastic Leukemia) OR (B-ALL) OR (B cell Leukemia,	4587

No.	Query	Results
	Lymphoblastic, Acute) OR (Leukemia, B Cell)	
#4	#1 AND #2 AND #3	57
#5	#4 in Clinical Trial	51
Embase		
#1	(Chimeric Antigen Receptor Therapy) OR (CAR T Cell Therapy) OR (CAR T)	33122
#2	(anti-CD19) OR (anti cd19)	8635
#3	(B cell acute lymphocytic leukaemia) OR (B cell acute Lymphoblastic Leukemia) OR (B-ALL)	94290
#4	#1 AND #2 AND #3	801
#5	#4 in Clinical Trial	215

Method S2. Inclusion/exclusion criteria

The inclusion criteria were:

1. Clinical trials that used anti-CD19 CAR T cells to treat B-ALL.

2. The study population included both children and adults.

3. The literature contained efficacy indicators (OS, PFS) and safety indicators.

The exclusion criteria were:

1. Non-clinical trial original literature, such as meta-analyses, reviews, retrospective studies, in vitro studies, non-original data (e.g., narrative reviews, editorials), and so on.

2. Studies with a small sample size (N<5).

3. Studies in languages other than English.

Method S3. Model building

Structural model establishment

Parametric survival models were utilized to examine survival data, such as OS and PFS, of patients with B-ALL who received anti-CD19 CAR T cell therapy. The survival model is associated with the hazard function h(t), which represents the instantaneous hazard at time t. Equation (S1) describes the relationship between the survival and hazard functions.

Survival model $$\mathrm{S(t)} = \exp\left(-\int_0^t h(t)dt\right) \quad \text{(S1)}$$

In Equation (S1), $\int_0^t h(t)dt$ is the cumulative risk from time 0 to time t, and S(t) is the survival rate at time t.

In order to choose the appropriate structural model, four hazard functions were assessed (Equations (S2–S5)). The hazard function in Equations (S2–S4) is determined by two parameters, λ and β, which represent the risk rate at time 0 and the regression coefficient of the risk rate, respectively, which changes over time. The hazard function in Equation (S5) adheres to the log-normal distribution, where μ and σ represent the median and standard deviation of the log-normal distribution, respectively.

Constant: $h(t)= \lambda$ (S2)

Gompert: $h(t)= \lambda\cdot\exp(\beta\cdot t)$ (S3)

Weibull: $h(t)= \lambda\cdot\exp(\beta\cdot\ln(t))$ (S4)

Lognormal: $$\mathrm{h(t)} = \frac{(\sigma t\sqrt{2\pi})^{-1}e^{(-\frac{1}{2}z^2)}}{1-\emptyset(z)}, \qquad Z = \frac{\ln(t)-\mu}{\sigma} \quad \text{(S5)}$$

The four hazard functions were selected based on the following criteria: the minimum value of the objective function value (OFV) provided by NONMEM software, the relative standard errors (RSEs) of the model parameter estimates, and the goodness of fit (GOF) plots.

Random effects model establishment

To account for the differences between observed values and model predicted values, the model was modified by adding inter-trial variability and residual error. The inter-trial

variability was incorporated into the model using an exponential form, as described in Equation (S6). The residual error was accounted for by the additive model, as explained in Equation (S7).

$$P_i = P_{pop} \times \exp(\eta_i) \quad \text{(S6)}$$

$$Obs_{i,j} = Pred_{i,j} + \mathrm{SE}_{i,j} \times \varepsilon_{i,j} \quad \text{(S7)}$$

$$SE_{\mathrm{i,j}} = \sqrt{\frac{Obs_{i,j} \times (1 - Obs_{i,j})}{N_{i,j}}} \quad \text{(S8)}$$

In Equation (S6), P_i is the individual value of the model parameter of trial i, P_{pop} is the population typical value of the model parameter, and η is the inter-trial variability of the model parameter, which conforms to a normal distribution with a mean of 0 and variance of ω_i^2. In Equation (S7), $Obs_{i,j}$ and $Pred_{i,j}$ are the observed and predicted survival data of trial i at time j, respectively. $\varepsilon_{i,j}$ is the residual error of trial i at time j, which conforms to a normal distribution with mean of 0 and variance of σ^2. $SE_{i,j}$ is the the standard error corresponds to the observed survival value, that is, the smaller the standard error is, the smaller the residual error. Equation (S8) is the formula for the standard error of survival value, where $N_{i,j}$ is the sample size of trial i at time j.

Covariate model establishment

The study investigated factors that could potentially affect the model parameters, such as age, proportion of males, proportion of patients with morphological relapse, median lines of previous therapies, proportion of primary refractory cases, proportion of patients who underwent prior allo-HSCT, and proportion of patients who received bridging allo-HSCT after CAR T cell therapy. Factors with missing data of 30% or more were not taken into consideration, while missing values for factors with less than 30% missing data were replaced with the median value. Categorical factors were incorporated into Equation (S9), while continuous factors were incorporated into Equation (S10).

$$h(t)_{final} = h(t)_{base} \times e^{(COV \times \theta_{\mathrm{cov}})} \quad \text{(S9)}$$

$$h(t)_{final} = h(t)_{base} \times e^{(COV - Cov_{median}) \times \theta_{cov}} \quad \text{(S10)}$$

In Equation (S9) and (S10), COV is the covariate value, COV_{median} is the median covariate, and θcov is the correction factor of the covariate for the model parameters. $h(t)_{base}$ is the hazard function when the categorical covariate is equal to 0 or continuous covariate is equal to COV_{median}.

The final covariates that were included in the model were selected using the forward introduction and reverse elimination methods. For the forward introduction method, a cut-off value of 3.84 ($p<0.05$) was used to determine if the decrease in the objective function value (OFV) was significant enough to include a covariate. Similarly, for the reverse elimination method, a cut-off value of 6.63 ($p<0.01$) was used to decide if the decrease in OFV was significant enough to remove a covariate from the model.

Method S4. Model assessment

After the final model was constructed, the model's goodness of fit was evaluated using Goodness of Fit (GOF) plots, which included scatterplots of the following: observation (OBS) vs. individual prediction (IPRED), OBS vs. population prediction (PRED), conditional weighted residual errors (CWRES) vs. PRED, and CWRES vs. time. The Visual Predictive Check (VPC) was employed to compare the model's predictions with the observed values and to assess the model's predictive performance. A sensitivity analysis was performed using the leave-one-out cross-validation method, where one set of study data was removed from the original dataset at a time, and the model parameters were estimated based on the resulting new dataset to evaluate the stability of the model. This was done by investigating the effect of each study on the model parameters. The robustness of the model was assessed using a nonparametric bootstrap with 1000 repetitions of the final model. The bootstrap median parameter values and percentile bootstrap 95% confidence interval (CI) were compared with the corresponding values estimated from the final model.

Table S2. The process of exploring the structural model of OS.

No	Model	OFV	Inclusion	Note
1	Constant model	-1867.896	No	
2	Gompertz model	-1995.866	No	
3	Weibull model	-1949.685	No	
4	Log normal model	-2155.221	Yes	The OFV is minimum, and the RSE% of the parameter is low.

Table S3. The process of exploring the structural model of PFS.

No	Model	OFV	Inclusion	Note
1	Constant model	-936.827	No	
2	Gompertz model	-1105.688	No	
3	Weibull model	-1089.958	No	
4	Log normal model	-1162.391	Yes	The OFV is minimum, and the RSE% of the parameter is low.

Table S4. Baseline characteristics of patients with B-cell acute lymphoblastic leukemia treated with anti-CD19 CAR T cells. Median(min-max)

	4-1BB	CD28	Overall
Number of trials(arm)	26(28)	5(7)	31(35)
Total sample size	700	228	928
Median age(year)	25.1(6.5-53.6)	44.0(13.5-45.0)	29.5(6.5-53.6)
Male (%)	58.0(37.5-92.9)	49.1(48.3-80.0)	57.3(37.5-92.9)
Median line of previous therapies	4(1-9)	3(2-4)	3(1-9)
Primary refractory (%)	8.0(0.0-55.0)	32.7(8.0-35.7)	10.6(0.0-55.0)
Disease burden (%)			
Median marrow blast	45.0(4.5-74.0)	61.0(4.0-63.0)	49.0(4.0-74.0)
Morphological relapse	80.0(28.6-100.0)	90.9(40.0-100.0)	82.5(28.6-100.0)
MRD-positive CR	10.0(0.0-62.5)	9.1(0.0-60.0)	10.0(0.0-62.5)
MRD-negative CR	0.0(0.0-54.1)	0.0(0.0-11.3)	0.00(0.0-54.1)
Prior allo-HSCT (%)	55.6(0.0-100.0)	38.8(20.0-44.0)	46.0(0.0-100.0)
Bridging allo-HSCT (%)	20.7(0.0-70.0)	50.0(18.2-82.6)	21.7(0.0-82.6)

MRD, minimal residual disease; CR, complete remission; allo-HSCT, allogeneic hematopoietic stem-cell transplantation.

Table S5. The list of included studies.

1. An F, Wang H, Liu Z, Wu F, Zhang J, Tao Q, et al. Influence of patient characteristics on chimeric antigen receptor T cell therapy in B-cell acute lymphoblastic leukemia. Nat Commun. 2020;11(1):5928.

2. Benjamin R, Graham C, Yallop D, Jozwik A, Mirci-Danicar OC, Lucchini G, et al. Genome-edited, donor-derived allogeneic anti-CD19 chimeric antigen receptor T cells in paediatric and adult B-cell acute lymphoblastic leukaemia: results of two phase 1 studies. Lancet (london, england). 2020;396(10266):1885 - 94.

3. Cao J, Wang G, Cheng H, Wei C, Qi K, Sang W, et al. Potent anti-leukemia activities of humanized CD19-targeted Chimeric antigen receptor T (CAR-T) cells in patients with relapsed/refractory acute lymphoblastic leukemia. American Journal of Hematology. 2018;93(7):851-8.

4. Curran KJ, Margossian SP, Kernan NA, Silverman LB, Williams DA, Shukla N, et al. Toxicity and response after CD19-specific CAR T-cell therapy in pediatric/young adult relapsed/refractory B-ALL. Blood. 2019;134(26):2361-8.

5. Dai H, Zhang W, Li X, Han Q, Guo Y, Zhang Y, et al. Tolerance and efficacy of autologous or donor-derived T cells expressing CD19 chimeric antigen receptors in adult B-ALL with extramedullary leukemia. OncoImmunology. 2015;4(11).

6. Frey NV, Shaw PA, Hexner EO, Pequignot E, Gill S, Luger SM, et al. Optimizing Chimeric Antigen Receptor T-Cell Therapy for Adults With Acute Lymphoblastic Leukemia. J Clin Oncol. 2020;38(5):415-22.

7. Gardner RA, Finney O, Annesley C, Brakke H, Summers C, Leger K, et al. Intent-to-treat leukemia remission by CD19 CAR T cells of defined formulation and dose in children and young adults. Blood. 2017;129(25):3322-31.

8. Gauthier J, Bezerra ED, Hirayama AV, Fiorenza S, Sheih A, Chou CK, et al. Factors associated with outcomes after a second CD19-targeted CAR T-cell infusion for refractory B-cell malignancies. Blood. 2021;137(3):323-35.

9. Ghorashian S, Kramer AM, Onuoha S, Wright G, Bartram J, Richardson R, et al. Enhanced CAR T cell expansion and prolonged persistence in pediatric patients with ALL treated with a low-affinity CD19 CAR. Nat Med. 2019;25(9):1408-14.

10. Gu R, Liu F, Zou D, Xu Y, Lu Y, Liu B, et al. Efficacy and safety of CD19 CAR T constructed with a new anti-CD19 chimeric antigen receptor in relapsed or refractory acute lymphoblastic leukemia. J Hematol Oncol. 2020;13(1):122.

11. Hay KA, Gauthier J, Hirayama AV, Voutsinas JM, Wu Q, Li D, et al. Factors associated with durable EFS in adult B-cell ALL patients achieving MRD-negative CR after CD19 CAR T-cell therapy. Blood. 2019;133(15):1652-63.

12. Heng G, Jia J, Li S, Fu G, Wang M, Qin D, et al. Sustained Therapeutic Efficacy of Humanized Anti-CD19 Chimeric Antigen Receptor T Cells in Relapsed/Refractory Acute Lymphoblastic Leukemia. Clin Cancer Res. 2020;26(7):1606-15.

13. Hiramatsu H, Adachi S, Umeda K, Kato I, Eldjerou L, Agostinho AC, et al. Efficacy and safety of tisagenlecleucel in Japanese pediatric and young adult patients with relapsed/refractory B cell acute lymphoblastic leukemia. Int J Hematol. 2020;111(2):303-10.

14. Hu Y, Wu Z, Luo Y, Shi J, Yu J, Pu C, et al. Potent Anti-leukemia Activities of Chimeric Antigen Receptor-Modified T Cells against CD19 in Chinese Patients with Relapsed/Refractory Acute Lymphocytic Leukemia. Clin Cancer Res. 2017;23(13):3297-306.

15. Hua J, Zhang J, zhang X, Wu X, Zhou L, Bao X, et al. Donor-derived anti-CD19 CAR T cells compared with donor lymphocyte infusion for recurrent B-ALL after allogeneic hematopoietic stem cell transplantation. Bone Marrow Transplantation. 2021;56(5):1056-64.

16. Jiang H, Liu L, Guo T, Wu Y, Ai L, Deng J, et al. Improving the safety of CAR-T cell therapy by controlling CRS-related coagulopathy. Ann Hematol. 2019;98(7):1721-32.

17. Kadauke S, Myers RM, Li Y, Aplenc R, Baniewicz D, Barrett DM, et al. Risk-Adapted Preemptive Tocilizumab to Prevent Severe Cytokine Release Syndrome After CTL019 for Pediatric B-Cell Acute Lymphoblastic Leukemia: A Prospective Clinical Trial. J Clin Oncol. 2021;39(8):920-30.

18. Liu P, Liu M, Lyu C, Lu W, Cui R, Wang J, et al. Acute Graft-Versus-Host Disease After Humanized Anti-CD19-CAR T Therapy in Relapsed B-ALL Patients After Allogeneic Hematopoietic Stem Cell Transplant. Frontiers in Oncology. 2020;10.

19. Ma F, Ho JY, Du H, Xuan F, Wu X, Wang Q, et al. Evidence of long-lasting anti-CD19 activity of engrafted CD19 chimeric antigen receptor-modified T cells in a phase I study targeting pediatrics with acute lymphoblastic leukemia. Hematol Oncol. 2019;37(5):601-8.

20. Maude SL, Frey N, Shaw PA, Aplenc R, Barrett DM, Bunin NJ, et al. Chimeric antigen receptor T cells for sustained remissions in leukemia. N Engl J Med. 2014;371(16):1507-17.

21. Maude SL, Laetsch TW, Buechner J, Rives S, Boyer M, Bittencourt H, et al. Tisagenlecleucel in Children and Young Adults with B-Cell Lymphoblastic Leukemia. N Engl J Med. 2018;378(5):439-48.

22. Myers RM, Li Y, Barz Leahy A, Barrett DM, Teachey DT, Callahan C, et al. Humanized CD19-Targeted Chimeric Antigen Receptor (CAR) T Cells in CAR-Naive and CAR-Exposed Children and Young Adults With Relapsed or Refractory Acute Lymphoblastic Leukemia. J Clin Oncol. 2021;39(27):3044-55.

23. Park JH, Rivière I, Gonen M, Wang X, Sénéchal B, Curran KJ, et al. Long-Term Follow-up of CD19 CAR Therapy in Acute Lymphoblastic Leukemia. N Engl J Med. 2018;378(5):449-59.

24. Shah BD, Bishop MR, Oluwole OO, Logan AC, Baer MR, Donnellan WB, et al. KTE-X19 anti-CD19 CAR T-cell therapy in adult relapsed/refractory acute lymphoblastic leukemia: ZUMA-3 phase 1 results. Blood. 2021;138(1):11-22.

25. Shah BD, Ghobadi A, Oluwole OO, Logan AC, Boissel N, Cassaday RD, et al. KTE-X19 for relapsed or refractory adult B-cell acute lymphoblastic leukaemia: phase 2 results of the single-arm, open-label, multicentre ZUMA-3 study. Lancet. 2021;398(10299):491-502.

26. Shah NN, Lee DW, Yates B, Yuan CM, Shalabi H, Martin S, et al. Long-Term Follow-Up of CD19-CAR T-Cell Therapy in Children and Young Adults With B-ALL. J Clin Oncol. 2021;39(15):1650-9.

27. Wan X, Yang X, Yang F, Wang T, Ding L, Song L, et al. Outcomes of Anti-CD19 CAR-T Treatment of Pediatric B-ALL with Bone Marrow and Extramedullary Relapse. Cancer research and treatment. 2021.

28. Wang J, Mou N, Yang Z, Li Q, Jiang Y, Meng J, et al. Efficacy and safety of humanized anti-CD19-CAR-T therapy following intensive lymphodepleting chemotherapy for refractory/relapsed B acute lymphoblastic leukaemia. British Journal of Haematology. 2020;191(2):212-22.

29. Wang T, Gao L, Hu X, Liu B, Chen J, Zhang W, et al. Chimeric Antigen Receptor-modified Donor Lymphocyte Infusion Improves the Survival of Acute Lymphoblastic Leukemia Patients With Relapsed Diseases After Allogeneic Hematopoietic Stem Cell Transplantation. J Immunother. 2019;42(3):81-8.

30. Yang F, Yang X, Bao X, Kang L, Zhou L, Wu X, et al. Anti-CD19 chimeric antigen receptor T-cells induce durable remission in relapsed Philadelphia chromosome-positive ALL with T315I mutation. Leukemia and Lymphoma. 2020;61(2):429-36.

31. Yang X, Dai H, Kang L, Qu C, Li Z, Yin J, et al. Donor origin CAR19 T cell infusion for B-ALL relapsed after allogeneic hematopoietic stem cell transplantation. Hematol Oncol. 2019;37(5):655-8.

Table S6. The details of the included studies.

Study	Year	Construct	ScFv	T-cell origin	Sample size	Median age	Male, n	Primary refractory, n	Prior allo-HSCT, n	Morphological relapse, n	Minimal residual disease positive CR, n	Minimal residual disease negative CR, n
An, F et al.	2020	4-1BB	FMC63	Atuo	47	22.0	23	3	9	9	32	15
Benjamin, R et al.	2020	4-1BB	4G7	Allo	21	22.0	NA	NA	13	13	15	6
Cao, J et al.	2018	4-1BB	FMC63	Atuo	17	20.3	NA	NA	NA	NA	16	1
Curran, K. J et al.	2019	CD28	FMC63	Atuo	25	13.5	NA	2	5	5	10	15
Dai, H et al.	2015	4-1BB	HM852952	Allo	9	35.0	4	NA	3	3	6	3
Frey, N. V et al.	2020	4-1BB	FMC63	Atuo	35	28.2	24	11	5	13	33	2
Gardner, R. A et al.	2017	4-1BB	FMC63	Atuo	43	12.3	23	3	28	28	28	8
Gauthier, J et al.	2021	4-1BB	FMC63	Atuo	14	45.0	9	NA	10	10	13	1
Ghorashian, S et al.	2019	4-1BB	CAT	Atuo	14	9.2	13	0	10	10	4	6
Gu, R et al.	2020	4-1BB	HI19α	Atuo	20	18.0	12	11	NA	NA	17	3
Hay, K. A et al.	2019	4-1BB	FMC63	Atuo	53	39.0	30	NA	23	23	34	19
Heng, G et al.	2020	4-1BB	FMC63	Atuo	10	16.0	7	NA	2	2	5	5
Hiramatsu, H et al.	2020	4-1BB	FMC63	Atuo	6	14.2	4	0	4	4	6	0

Study	Year	Construct	ScFv	T-cell origin	Sample size	Median age	Male, n	Primary refractory, n	Prior allo-HSCT, n	Morphological relapse, n	Minimal residual disease positive CR, n	Minimal residual disease negative CR, n
Hu, Y et al.	2017	4-1BB	FMC63	Atuo	15	35.6	9	1	5	5	13	1
Hua, J et al.	2021	4-1BB	FMC63	Allo	13	30.0	5	0	13	13	NA	NA
Jiang, H et al.	2019	4-1BB	NA	Atuo	53	31.9	NA	NA	2	2	33	14
Kadauke, S et al.	2021	4-1BB	FMC63	Atuo	70	11.2	41	14	25	25	26	17
Liu, P et al.	2020	4-1BB	NA	Allo	13	29.5	NA	0	13	13	13	0
Ma, F et al.	2019	4-1BB	FMC63	Atuo	10	6.5	4	1	0	0	9	1
Maude, S. L et al.	2014	4-1BB	FMC63	Atuo	30	14.0	18	3	18	18	24	1
Maude, S. L et al.	2018	4-1BB	FMC63	Atuo	75	11.0	43	6	46	46	74	1
Myers, R. M et al.	2021	4-1BB	Humanized	Atuo	74	11.5	49	9	34	34	22	12
Park, J. H et al.	2018	CD28	SJ25C1	Atuo	53	44.0	NA	12	19	19	27	20
Shah, B. D et al.	2021	CD28	FMC63	Atuo	55	40.0	33	18	23	23	50	5
Shah, B. D et al.	2021	CD28	FMC63	Atuo	45	45.0	22	16	NA	NA	45	0
Shah, N. N et al.	2021	CD28	FMC63	Atuo	50	13.5	40	11	22	22	33	17
Wan, X et al.	2021	4-1BB	FMC63	Atuo,A	8	7.9	3	NA	NA	NA	3	5

Study	Year	Construct	ScFv	T-cell origin	Sample size	Median age	Male, n	Primary refractory, n	Prior allo-HSCT, n	Morphological relapse, n	Minimal residual disease positive CR, n	Minimal residual disease negative CR, n
				llo								
Wang, J et al.	2020	4-1BB	Humanized	Atuo	23	39.3	14	NA	5	5	23	0
Wang, T et al.	2019	4-1BB	FMC63	Atuo	5	31.0	2	NA	5	5	3	0
Yang, F et al.	2020	4-1BB	FMC63	Atuo, Allo	7	39.4	4	0	5	5	5	2
Yang, X et al.	2019	4-1BB	NA	Allo	15	34	7	NA	15	15	15	0

NA, not available; Auto, Autologous; Allo, Allogeneic; scFv, single chain variable fragment. CR, complete remission.

Table S7. Secondary and safety outcomes of the included studies

Study	Overall remission, n	Minimal residual disease negative, n	cytokine release syndrome, n	Grade≥3 cytokine release syndrome, n	Neurotoxicity, n	Grade≥3 Neurotoxicity, n	Cytokine release syndrome grading criteria	Subsequent allo-HSCT, n
An, F et al. 2020	38	37	39	11	NA	1	NCI CTCAE 4.03	10
Benjamin, R et al. 2020	14	10	19	3	8	0	Lee	13
Cao, J et al. 2018	13	11	17	4	1	NA	Lee	4
Curran, K. J et al. 2019	18	16	20	4	18	7	Lee	15
Dai, H et al. 2015	4	2	4	3	2	0	NCI CTCAE 3.0	0
Frey, N. V et al. 2020	24	19	33	25	14	2	Penn	9
Gardner, R. A et al. 2017	40	40	40	10	21	9	NCI CTCAE 4.03	11
Gauthier, J et al. 2021	3	2	10	1	3	2	Lee	1
Ghorashian, S et al. 2019	12	12	13	0	7	1	Lee	0
Gu, R et al. 2020	18	18	19	9	13	8	Lee	14
Hay, K. A et al. 2019	45	45	40	10	NA	12	Lee	18
Heng, G et al. 2020	10	10	10	4	4	NA	MSKCC	2
Hiramatsu, H et al. 2020	4	4	5	5	1	NA	Penn	4
Hu, Y et al. 2017	12	12	10	6	5	NA	Lee	4
Hua, J et al. 2021	9	8	8	3	0	0	Lee	0
Jiang, H et al. 2019	47	43	53	19	8	NA	Lee	NA
Kadauke, S et al. 2021	68	67	52	12	19	4	Penn	6
Liu, P et al. 2020	13	11	13	4.3	4.3	NA	NCI CTCAE 4.03	0
Ma, F et al. 2019	8	8	10	4	6	3	NCI CTCAE 4.03	0
Maude, S. L et al. 2014	27	22	30	8	13	NA	Penn	3
Maude, S. L et al. 2018	61	61	58	35	30	10	Penn	17

Study	Overall remission, n	Minimal residual disease negative, n	cytokine release syndrome, n	Grade≥3 cytokine release syndrome, n	Neurotoxicity, n	Grade≥3 Neurotoxicity, n	Cytokine release syndrome grading criteria	Subsequent allo-HSCT, n
Myers, R. M et al. 2021	61	58	61	11	29	3	Penn	5
Park, J. H et al. 2018	44	32	45	14	24	22	MSKCC	16
Shah, B. D et al. 2021	39	39	49	13	33	14	Lee	10
Shah, B. D et al. 2021*	31	NA	42	14	35	17	Lee	31
Shah, N. N et al. 2021	31	28	35	9	10	4	Lee	21
Wan, X et al. 2021	8	6	7	2	NA	NA	Lee	2
Wang, J et al. 2020	19	18	23	5	0	0	Lee	5
Wang, T et al. 2019	5	5	5	1	NA	NA	Penn	0
Yang, F et al. 2020	6	3	4	1	NA	NA	Lee	2
Yang, X et al. 2019	11	8	4	NA	NA	NA	NA	NA

* Shah BD, 2021, Lancet. allo-HSCT, allogeneic hematopoietic stem-cell transplantation; NA, not available; Penn, University of Pennsylvania grading system; NCI, National Cancer Institute; CTCAE, Common Terminology Criteria for Adverse Events; MSKCC, Memorial Sloan Kettering Cancer Center grading system; ASTCT, American Society for Transplantation and Cellular Therapy grading system.

Table S8. Risk assessment for inclusion in the studies.

Study	Domain 1: Confounding	Domain 2: Selection	Domain 3: Classification of intervention	Domain 4: Deviation from interventions	Domain 5: Missing data	Domain 6: Measurement of outcomes	Domain 7: Selection of reported result	ROBINS-I overall
An, F et al. 2020	Low	Low	Low	Low	Low	Moderate	Low	Moderate
Benjamin, R et al. 2020	Moderate	Low	Low	Moderate	Low	Moderate	Low	Moderate
Cao, J et al. 2018	Low	Low	Low	Moderate	Low	Moderate	Low	Moderate
Curran, K. J et al. 2019	Low	Low	Low	Moderate	Low	Moderate	Moderate	Moderate
Dai, H et al. 2015	Serious	Low	Low	Moderate	Low	Moderate	Low	Serious
Frey, N. V et al. 2020	Low	Low	Low	Low	Low	Low	Moderate	Moderate
Gardner, R. A et al. 2017	Low	Low	Low	Low	Low	Moderate	Low	Moderate
Gauthier, J et al. 2021	Moderate	Low	Moderate	Low	Low	Moderate	Serious	Serious
Ghorashian, S et al. 2019	Low	Low	Low	Low	Low	Moderate	Low	Moderate
Gu, R et al. 2020	Low	Low	Low	Low	Low	Moderate	Low	Moderate
Hay, K. A et al. 2019	Low	Low	Low	Moderate	Low	Low	Serious	Serious
Heng, G et al. 2020	Moderate	Low	Low	Moderate	Moderate	Moderate	Low	Moderate
Hiramatsu, H et al. 2020	Moderate	Low	Low	Low	Moderate	Moderate	Moderate	Moderate
Hu, Y et al. 2017	Low	Low	Low	Low	Low	Low	Low	Low
Hua, J et al. 2021	Low	Low	Low	Low	Low	Low	Low	Low
Jiang, H et al. 2019	Serious	Low	Low	Low	Low	Low	Moderate	Serious
Kadauke, S et al. 2021	Low	Low	Low	Low	Moderate	Low	Low	Moderate
Liu, P et al. 2020	Low	Low	Low	Low	Serious	Low	Low	Serious
Ma, F et al. 2019	Low	Low	Low	Low	Low	Low	Low	Low
Maude, S. L et al. 2014	Low	Low	Low	Low	Low	Low	Low	Low

Maude, S. L et al. 2018	Low	Low	Low	Low	Low	Low	Low	Low
Myers, R. M et al. 2021	Low	Low	Low	Moderate	Low	Moderate	Moderate	Moderate
Park, J. H et al. 2018	Low	Low	Low	Low	Low	Low	Low	Low
Shah, B. D et al. 2021	Low	Low	Low	Low	Low	Moderate	Moderate	Moderate
Shah, B. D et al. 2021*	Moderate	Low	Low	Low	Moderate	Moderate	Low	Moderate
Shah, N. N et al. 2021	Low	Low	Low	Low	Low	Low	Low	Low
Wan, X et al. 2021	Low	Low	Low	Low	Low	Moderate	Low	Moderate
Wang, J et al. 2020	Moderate	Low	Moderate	Low	Moderate	Low	Moderate	Moderate
Wang, T et al. 2019	Moderate	Low	Low	Low	Low	Moderate	Moderate	Moderate
Yang, F et al. 2020	Moderate	Low	Low	Low	Low	Low	Moderate	Moderate
Yang, X et al. 2019	Moderate	Low	Low	Low	Low	Low	Moderate	Moderate

* Shah BD, 2021, Lancet.

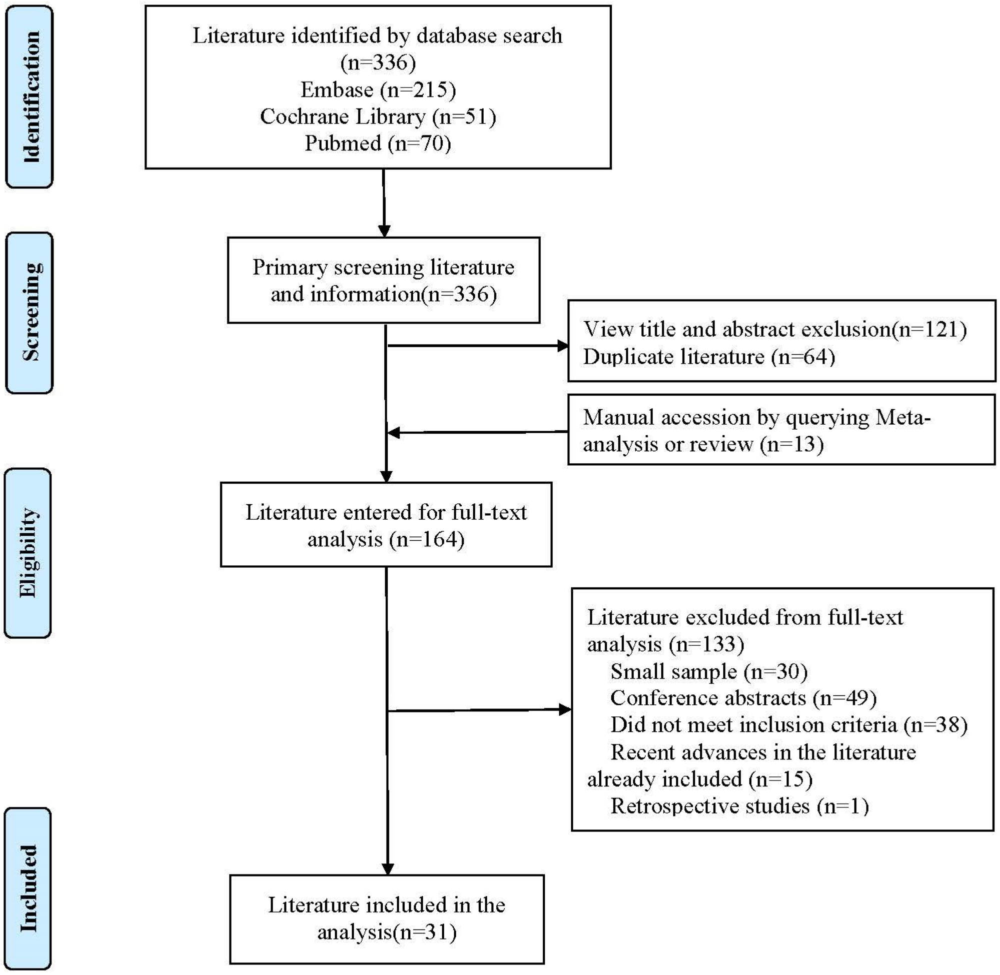

Figure S1. Flow chart of study selection.

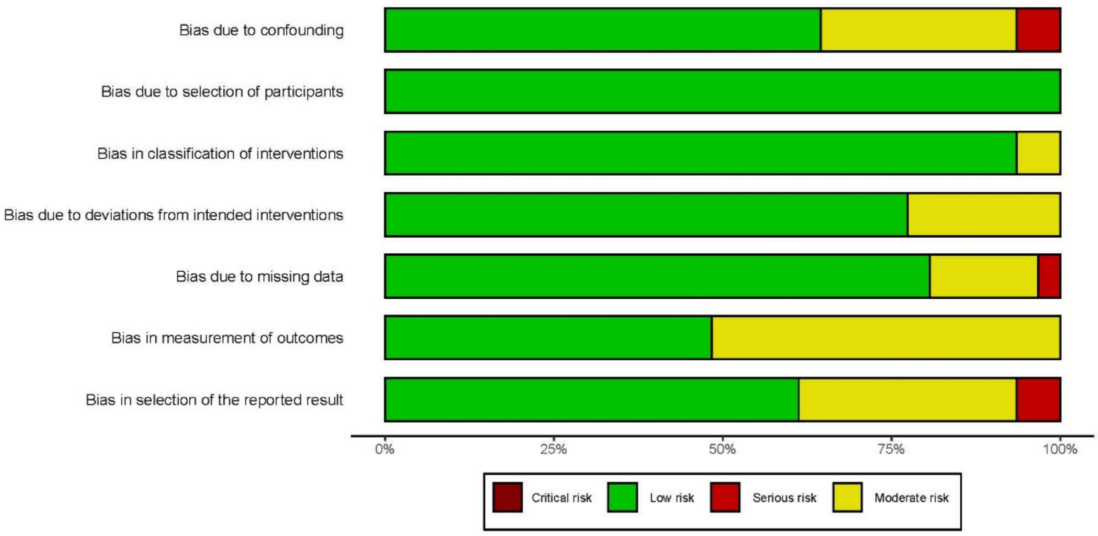

Figure S2. The summary risk of literature assessment.

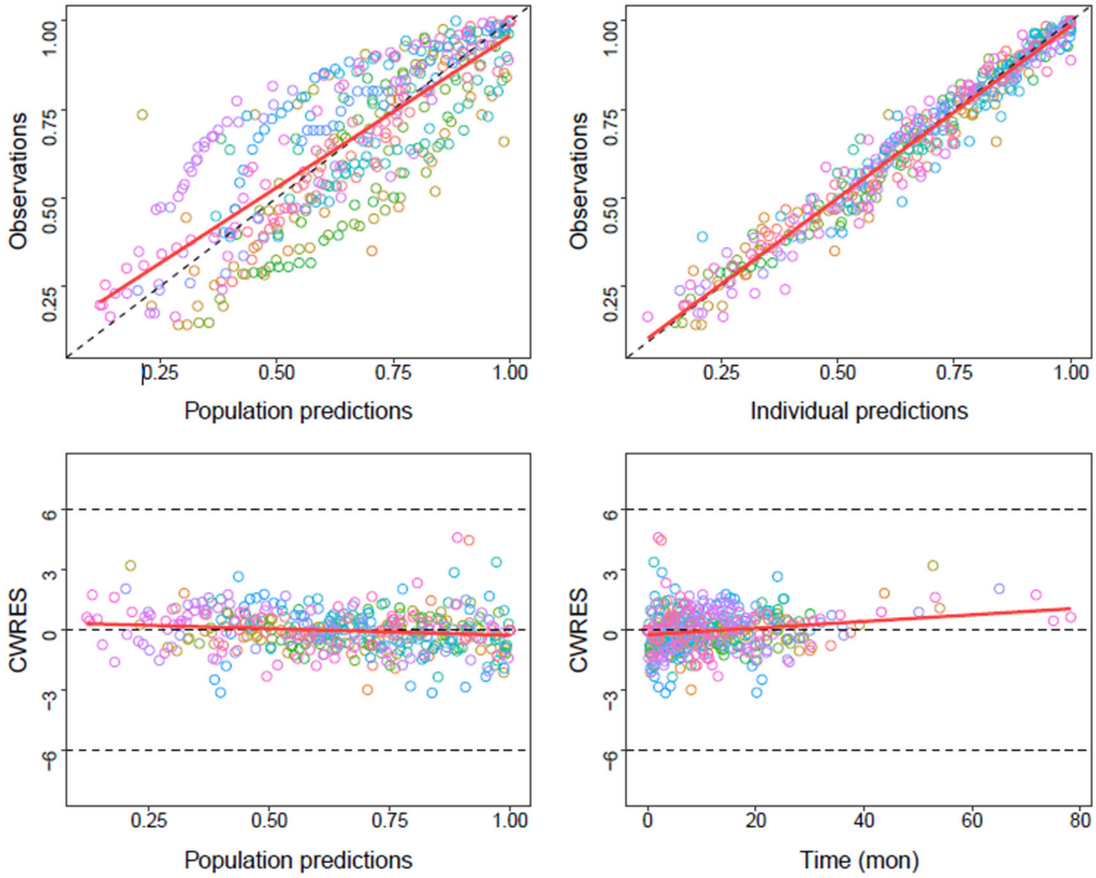

Figure S3. The goodness-of-fit plots of OS model. The top-left graph displays the population predicted (PRED) values against the observed values (OBS), while the top-right graph shows the individual predicted (IPRED) values against OBS. The bottom-left graph presents the conditional weighted residuals (CWRES) against PRED, and the bottom-right graph shows CWRES against time. The top-left and top-right graphs have dashed and solid lines that represent the diagonal and fitting lines, respectively, while the bottom-left and bottom-right graphs have dashed and solid lines that represent the 0 and ±6 lines and fitting lines, respectively. The colors of the points indicate the test groups.

The results indicate that OBS values are evenly distributed on both sides of the diagonal for PRED and IPRED, and the fitting line nearly coincides with the diagonal. Most CWRES values are distributed approximately evenly around the zero (0) line within six, and the fitting lines of CWRES versus PRED and CWRES versus time nearly coincide with the zero (0) line. These results suggest that the model fits well, and there is no noticeable bias.

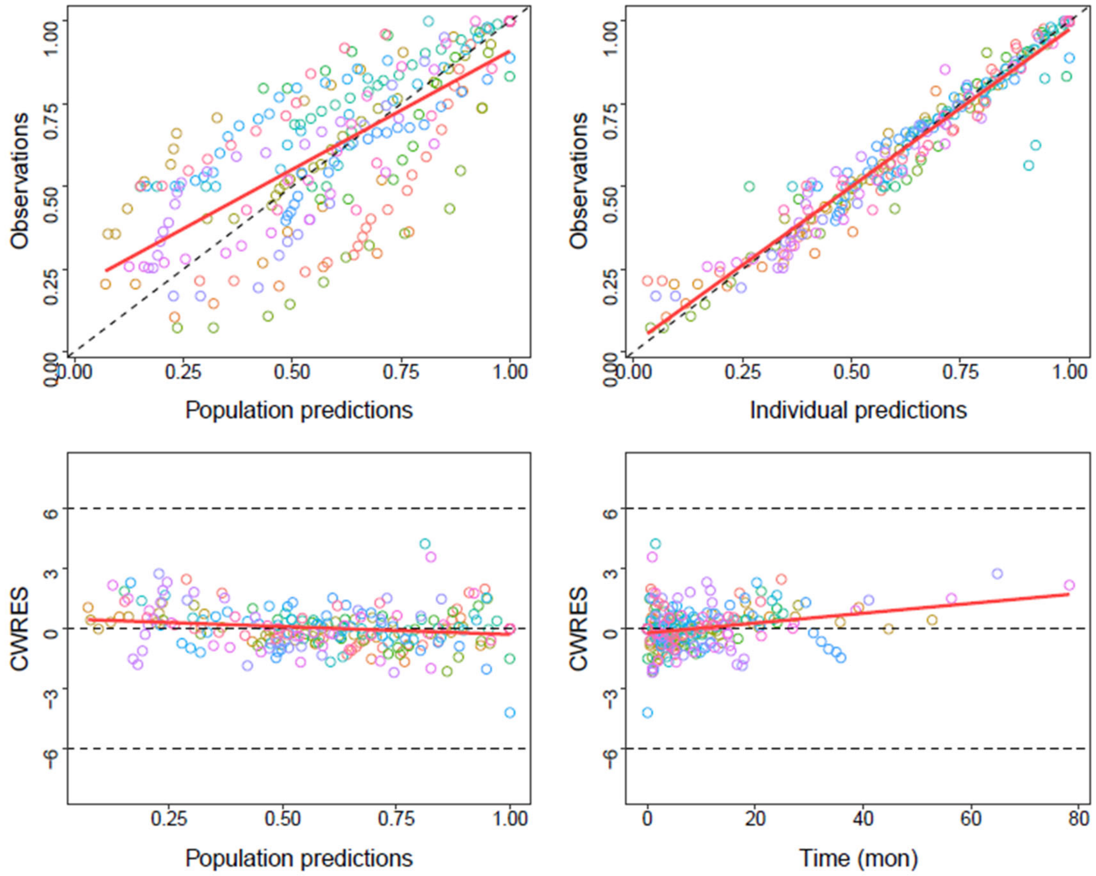

Figure S4. The goodness-of-fit plots of PFS model. The top-left graph displays the population predicted (PRED) values against the observed values (OBS), while the top-right graph shows the individual predicted (IPRED) values against OBS. The bottom-left graph presents the conditional weighted residuals (CWRES) against PRED, and the bottom-right graph shows CWRES against time. The top-left and top-right graphs have dashed and solid lines that represent the diagonal and fitting lines, respectively, while the bottom-left and bottom-right graphs have dashed and solid lines that represent the 0 and ±6 lines and fitting lines, respectively. The colors of the points indicate the test groups.

The results indicate that OBS values are evenly distributed on both sides of the diagonal for PRED and IPRED, and the fitting line nearly coincides with the diagonal. Most CWRES values are distributed approximately evenly around the zero (0) line within six, and the fitting lines of CWRES versus PRED and CWRES versus time nearly coincide with the zero (0) line. These results suggest that the model fits well, and there is no noticeable bias.

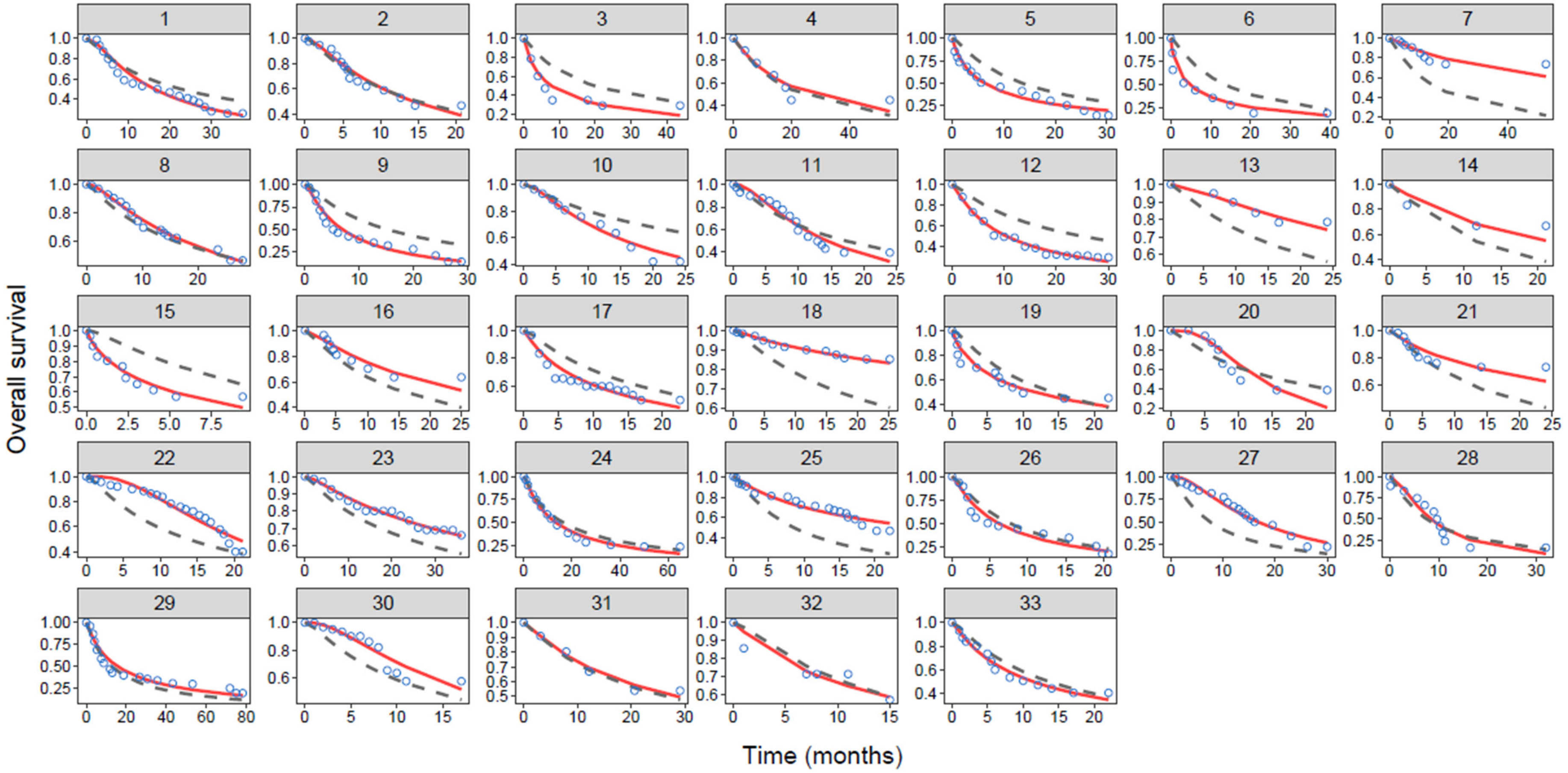

Figure S5. Individual trial fitting graph of OS model. The dots represent the observed values, the dotted lines represent the population predicted values, and the red lines represent the individual predicted values. Based on the results, it appears that the model fits well, as the individual predicted values are very similar to the observed values.

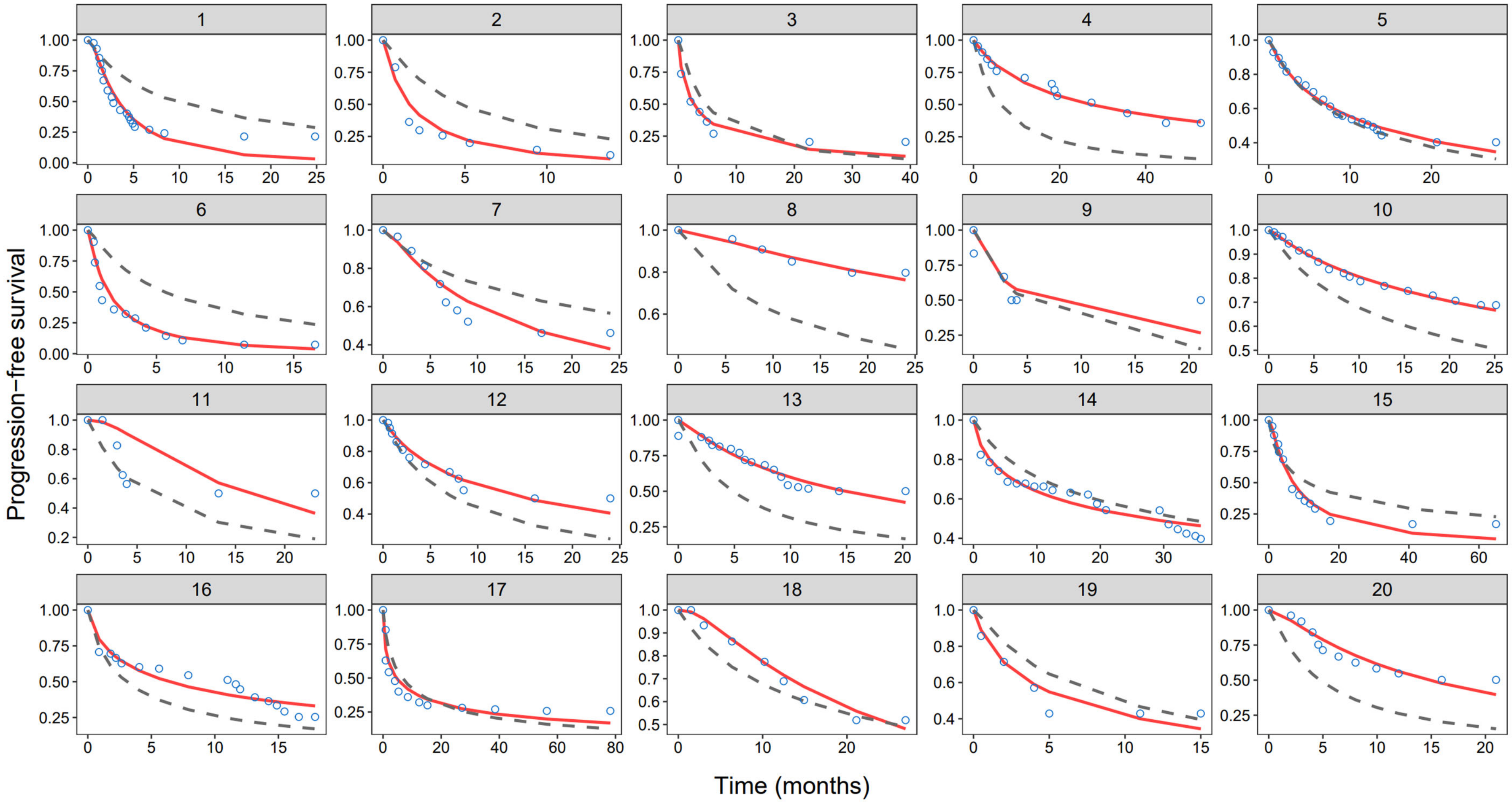

Figure S6. Individual trial fitting graph of PFS model. The dots represent the observed values, the dotted lines represent the population predicted values, and the red lines represent the individual predicted values. Based on the results, it appears that the model fits well, as the individual predicted values are very similar to the observed values.

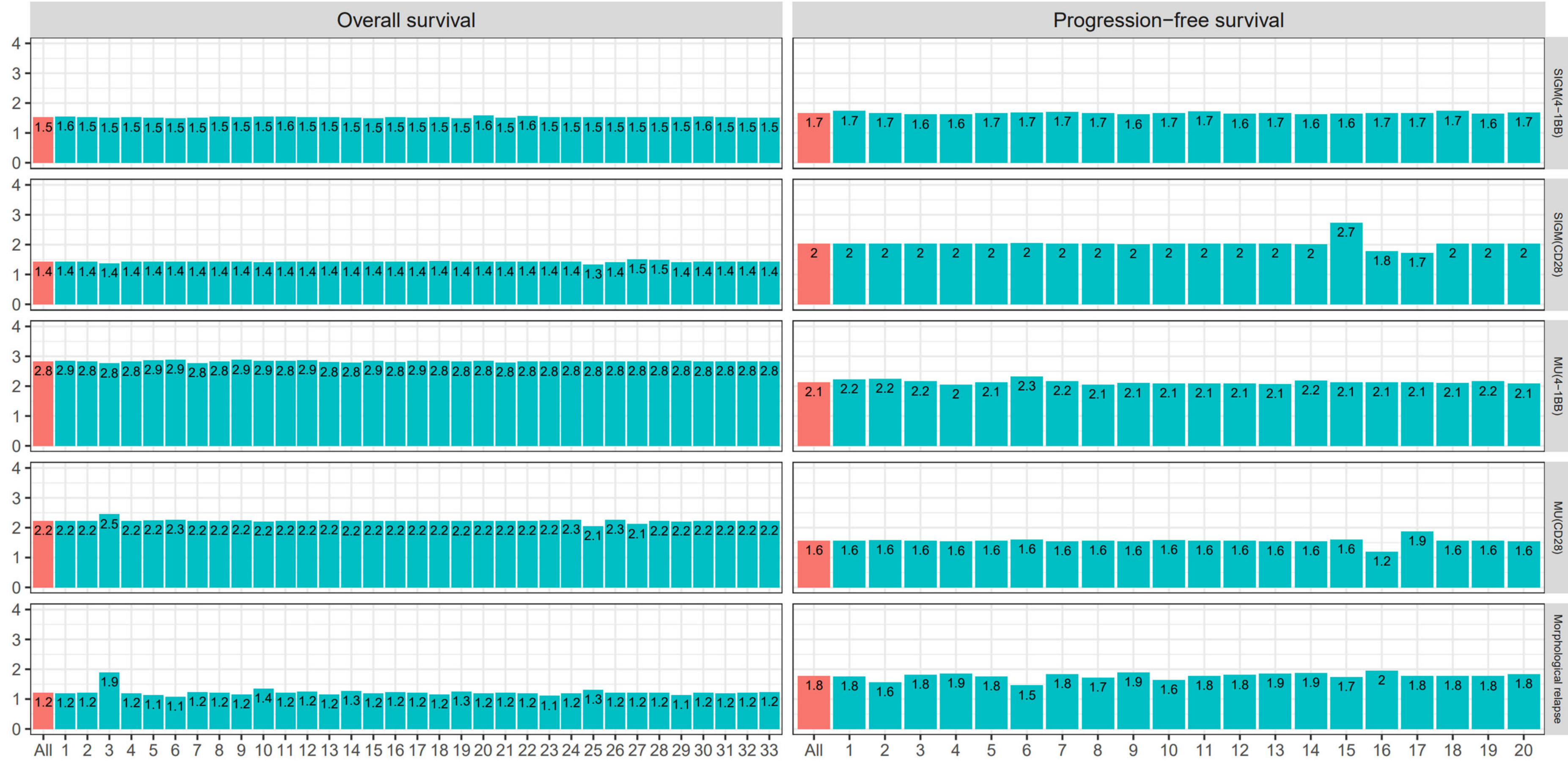

Figure S7. Sensitivity analysis of model parameters by the leave-one-out cross validation. The horizontal axis represents the test number excluded from the complete data, and the vertical axis represents the estimated value of the model parameters. The red bar represents the parameters obtained from the complete data.

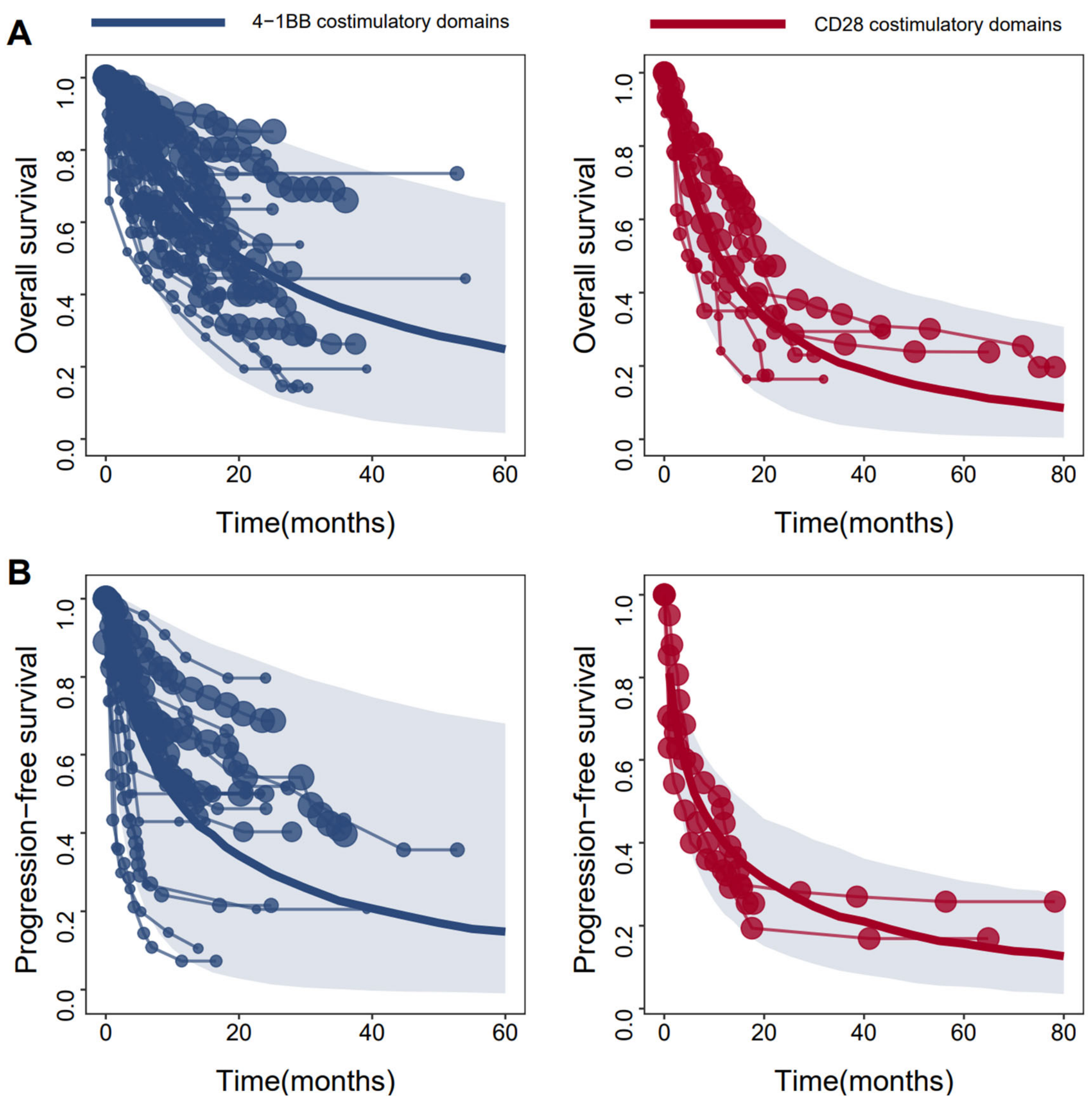

Figure S8. Visual prediction check of the final model of OS (A) and PFS (B). The points in the graph represent the measured values, and the size of each point is proportional to the number of patients in each trial. Points that lie on the same line correspond to the same trial. The shaded area in the graph represents the 95th percentile of model prediction, while the solid line represents the median value. Based on the figure, it is evident that most of the measured points fall within the 95% CI predicted by the model. This suggests that the model has good predictive ability.

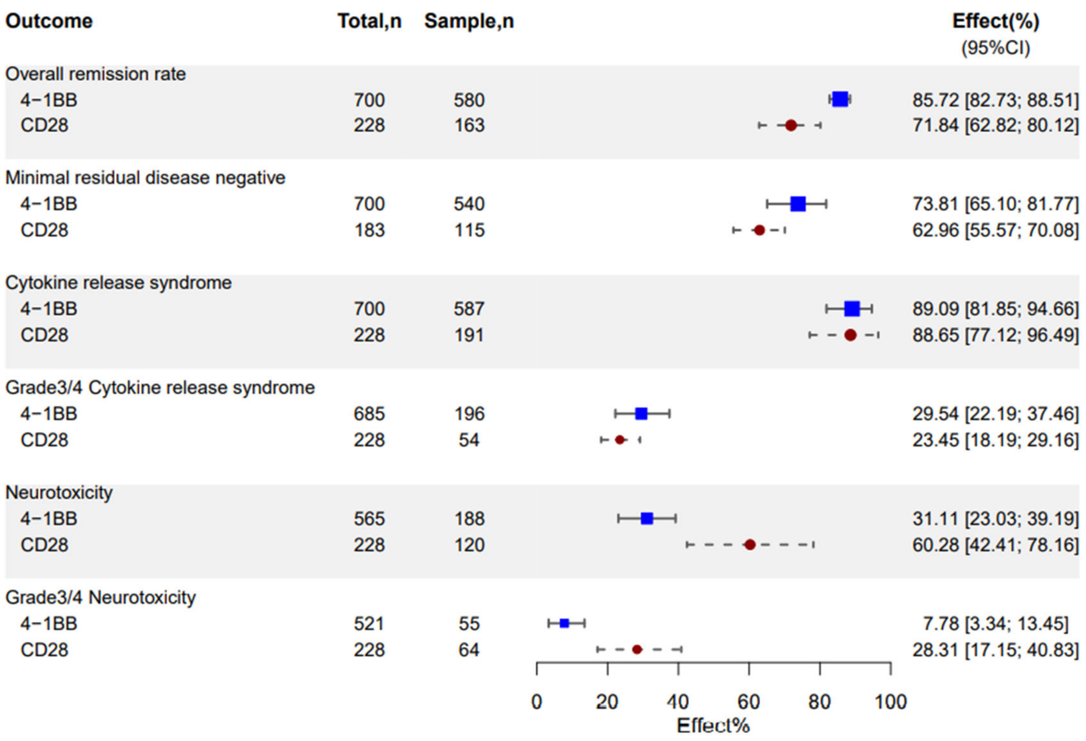

Figure S9. Forest plot comparing secondary and safety outcomes of different costimulatory domains, with box/point size proportional to sample size.

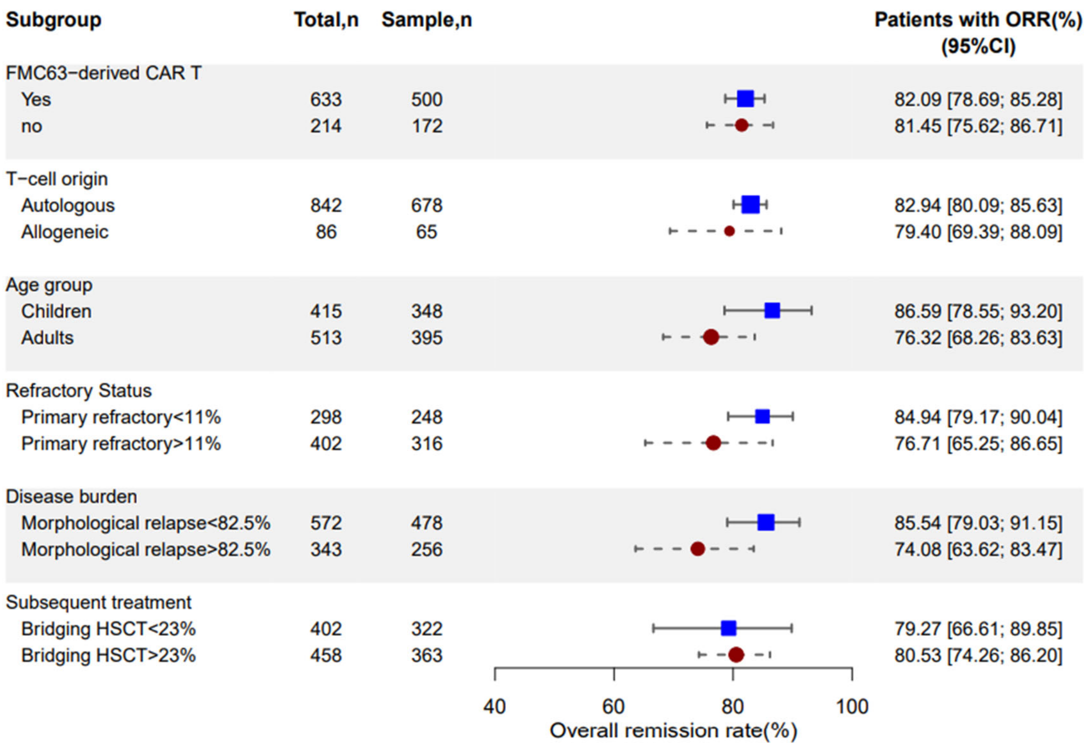

Figure S10. Subgroup analysis of ORR. The size of the box/point is proportional to the sample size. Adults are patients aged ≥18 years and children are patients aged <18 years.

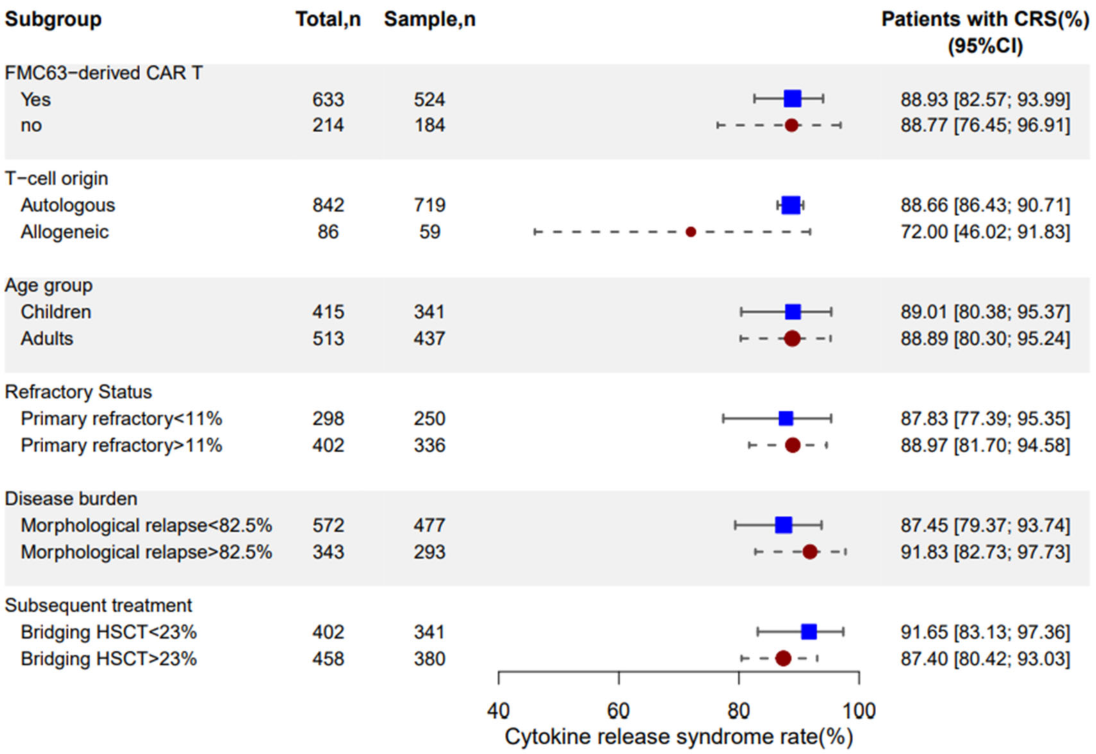

Figure S11. Subgroup analysis of CRS. The size of the box/point is proportional to the sample size. Adults are patients aged ≥18 years and children are patients aged <18 years.

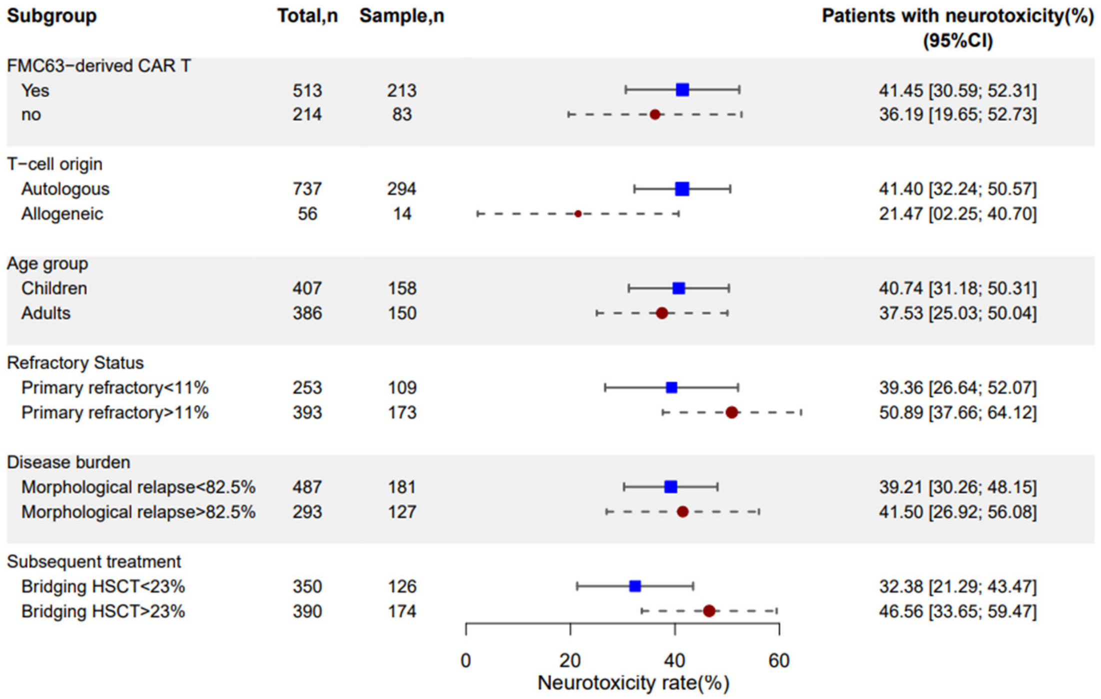

Figure S12. Subgroup analysis of neurotoxicity. The size of the box/point is proportional to the sample size. Adults are patients aged ≥18 years and children are patients aged <18 years.
